# Fed-XLM-R: a privacy-preserving federated framework with adapter-scoped differential privacy for mental health triage-level intent classification on resource-constrained edge devices

**DOI:** 10.3389/fdgth.2026.1856286

**Published:** 2026-09-09

**Authors:** Karthiga M, Emerson Raja Joseph, Subhash Patil, K. Saranya

**Affiliations:** 1 Department of Computer Science and Engineering, Bannari Amman Institute of Technology, Sathyamangalam, Erode, Tamilnadu, India; 2 Postdoctoral Researcher, Multimedia University, Melaka, Malaysia; 3 Faculty of Engineering and Technology, Centre for Advanced Analytics COE of Artificial Intelligence, Multimedia University, Melaka, Malaysia; 4 Professor and Head of Surgery Department, International Medical University, Management and Science University, Federal Territory of Kuala Lumpur, Malaysia

**Keywords:** adapter fine-tuning, cross-lingual transfer, differential privacy, DP-SGD, edge deployment, federated learning, FedProx, INT8 quantization

## Abstract

**Introduction:**

Mental health triage-level intent classification in linguistically diverse and resource-constrained clinical environments requires models that simultaneously meet strict patient data privacy regulations, cross-lingual generalization to low-resource languages, and real-time inference on edge hardware. No existing federated NLP system has jointly demonstrated this combination. This work proposes Fed-XLM-R, a unified federated learning framework designed to close that gap.

**Methods:**

Fed-XLM-R integrates XLM-RoBERTa with lightweight bottleneck adapter layers, adapter-scoped differentially private stochastic gradient descent (DP-SGD), proximal regularization for straggler-robust aggregation (FedProx), and INT8 post-training quantization for edge deployment. Gradient perturbation is confined exclusively to the 0.87% trainable adapter subspace rather than the full 270-million-parameter model. The framework was evaluated in a simulated 10-client federation on a public benchmark, with intent classification trained and evaluated on English data. A cross-lingual diagnostic was additionally run on the XNLI benchmark across five languages (English, Spanish, Hindi, Swahili, Arabic) against an mBERT baseline, and canary phrase memorization analysis was conducted across all 50 federated rounds to assess data extraction risk.

**Results:**

At the clinically meaningful privacy budget of *ε* = 1.0 (*δ* = 10^−5^, Fed-XLM-R achieves 96% accuracy retention, yielding an overall accuracy of 0.923, F1-score of 0.918, and AUC-ROC of 0.956, within 0.5% of a centralized baseline while providing formal differential privacy guarantees. Canary phrase analysis confirms the DP-SGD mechanism prevents training data extraction. The XLM-R backbone retains strong discriminative capacity in low-resource languages relative to mBERT on the XNLI diagnostic. Adapter-only parameter transmission reduces cumulative communication cost by 40.7-fold at the 85% accuracy threshold, at a per-round volume compatible with standard mobile data networks. INT8 quantization reduces inference latency from 1,974 ms to 523 ms on low-end CPU hardware, a 3.8-fold speedup.

**Discussion:**

These findings characterize the mechanism's behavior under controlled conditions in a simulated federation rather than demonstrating field deployment. The cross-lingual results support the feasibility of future multilingual extension but do not themselves constitute multilingual intent classification. The three-category label scheme (Anxiety/Depression/Normal) supports triage-level intent routing but has not been validated against standardized clinical instruments; the system is not proposed as a clinical diagnostic or screening tool. Overall, Fed-XLM-R demonstrates that privacy-preserving, communication-efficient, and edge-deployable federated NLP is achievable without substantial accuracy loss, offering a practical pathway toward privacy-compliant mental health triage systems pending real-world and multilingual validation.

## 1 Introduction

Mental health disorders, especially anxiety and depression, are a growing concern for the global population. According to the World Health Organization (WHO), there are over one billion individuals worldwide that suffer from some form of mental health disorder, yet less than half of those individuals receive any form of treatment for their condition. Many of the regions in the world with the highest rates of mental health disorders, such as parts of Africa, Asia, and the Middle East, have a near-complete lack of mental health chatbots that cater to their languages, such as Swahili, Hindi, and Arabic. Though mental health chatbots have been proposed as a solution to increase the number of individuals that are screened for mental health disorders, there are two main reasons that these bots are currently not deployed in mental health clinics or facilities: (i) The privacy rules (e.g., GDPR and HIPAA) that regulate patient data sharing create tight restrictions on the sharing of patient sensitive health information, preventing patient data from leaving the institution and patient device (7), and (ii) most natural language processing models that currently exist have been trained using English-language text and exhibit a significant drop in performance when applied to languages with less available textual data, such as Swahili, Hindi, and Arabic (23, 24).

Federated learning has emerged as a principled response to the data-privacy constraint by allowing model training to proceed across distributed clinical nodes without centralizing raw patient records (1, 10). Recent studies have extended federated learning to medical image analysis (8, 9), cardiovascular screening (12), rare disease prediction (1), and clinical text generation (3). However, rather than treating privacy and multilingual coverage as jointly optimized requirements, these systems usually treat them as distinct design objectives. Distributing training alone does not provide a formal privacy guarantee (6, 38), and a federated system that transmits complete model parameters at each communication round can still leak private information through gradient inference attacks (41). The mathematical assurance that no specific training record can be recreated from model updates is provided by Differential Privacy (DP), which is implemented through per-sample gradient clipping and calibrated noise injection (36, 37). However, integrating DP with federated NLP training presents a unique problem: unless the trainable parameter space is strictly constrained, adding Gaussian noise to gradients of a transformer with 270 million parameters significantly reduces accuracy (21, 40).

This limitation is directly addressed by parameter-efficient fine-tuning through bottleneck adapter layers (28). In comparison to full-model fine-tuning, the sensitivity of gradient updates to DP noise is reduced by more than two orders of magnitude by freezing the base transformer weights and training only compact adapter modules (roughly 2.4 million parameters in the suggested setting) (11, 13). The fundamental layout of the suggested framework is inspired by this realization. Standard multilingual BERT (mBERT) has been demonstrated to yield F1-scores of 0.60 to 0.72 on Swahili and Hindi clinical classification tasks (23, 33), which are significantly below the performance thresholds necessary for trustworthy triage-level intent classification. This indicates that cross-lingual coverage for low-resource languages is still a separate bottleneck. The core of the suggested system is XLM-RoBERTa, which has been pretrained on 2.5 TB of multilingual text and offers significantly stronger representations for these languages (33).

Another issue with federated clinical deployment is device heterogeneity (18). Slower clients (stragglers) that finish fewer local training steps than planned destabilize the FedAvg aggregation step and lower round completion rates to as low as 55% under high-straggler conditions when client devices span hospital servers, mid-range laptops, and community health smartphones (20, 22). FedProx's proximal regularization term maintains completion rates above 90% even in straggler events by bounding client drift regardless of the number of local steps each client completes (22).

In light of this, the current work suggests Fed-XLM-R is an integrated framework for mental health NLP which addresses privacy, cross-lingual backbone transferability, communication efficiency and edge deployability all at once. The following particular contributions are made by the framework:

- A federated adapter-tuning strategy that restricts DP-SGD gradient perturbation to the 0.87% trainable adapter subspace, achieving 96% accuracy retention at the clinically meaningful privacy budget of *ε* = 1.0 (21).
- A joint DP-FedProx local update rule that simultaneously enforces differential privacy at the sample level and limits client drift at the round level, a combination not previously demonstrated in federated clinical NLP (22, 40).
- An XLM-R model for cross-lingual diagnostics of language-representation on XNLI across 5 languages (English, Spanish, Hindi, Swahili, Arabic) that demonstrates the discriminative capacity of the XLM-R backbone model over mBERT, prompting, but not confirmation, of multilingual clinical extension (23, 33).
- The cumulative communication cost is reduced by 40.7 times in the 85% accuracy checkpoint using the adapter only parameter transmission method, resulting in the amount of communication per round that is comparable to that of conventional mobile communications networks (17, 19).
- In addition, the INT8 post training quantization technique has been demonstrated for reducing measured inference latency from 1,974 ms to 523 ms, showing the potential of real-time inference on edge class hardware under single inference requests.

To prevent ambiguity, we define the scope of the proposed system at the beginning: Fed-XLM-R is a text classification system for triage level mental health intent classification of an input utterance into one of the three symptomatic intent categories (Anxiety, Depression, Normal). The intent classification at the triage level represents a broad symptomatic class of an input utterance and is used to support initial routing or prioritisation, and differs from clinical diagnosis or screening, which should be validated against standardized instruments (e.g., the PHQ-9 or GAD-7) administered by an appropriately trained clinician. Future work identified for clinical deployment includes correlation with such instruments and expert label validation. It doesn't elicit therapeutic or conversational answers (5). The conversational corpora used in this work are only input text for representation learning and classification; there is no component of the framework that involves a decoder, response-generation or dialogue-generation. The remainder of this paper is organized as follows. Section 2 reviews related work. Section 3 presents the proposed methodology. Section 4 reports experimental results and discussion. Section 5 concludes.

## 2 Related work

### 2.1 Federated learning in healthcare NLP

Federated learning has become much more common in healthcare text analysis in the last few years. Kunjumohamad and Govindaraj (1) proposed a federated method of predicting multiorganic rare diseases. Their study showed that the diagnostic utility can be maintained in decentralized training without the need to centralize clinical records. Lal et al. (2) also used large language models and federated learning together to make medical insights with pipelines driven by Ollama. They demonstrated that LLMs can be used in sensitive privacy-related clinical workflows. Ma et al. (3) touched upon the relevant topic using the concept of a text generation based on LLM to promote personal federated datasets. This approach can address the issue of the lack of data, which is typical of clinical NLP, particularly in a low-resource setting. All of these studies confirm the practicability of federated NLP in healthcare, but they do not look at cross-lingual implementation or formal differential privacy.

Kanauzia et al. (10) performed an in-depth analysis of federated learning to protect privacy in electronic healthcare. They examined the existing state of affairs in the imaging, signal processing and text fields. Their review shows that three issues remain consistent, namely: the lack of formal privacy guarantees in most federated health systems, the lack of coverage of non-English clinical languages, and the high cost of communication of full model parameters in each iteration. This is a piece of work that aims to address all the three problems. Poojari (7) investigated the privacy-sensitive generative AI in the medical field by using federated learning. They claimed that the necessity of decentralized training arrangements is due to the regulatory pressure of HIPAA and GDPR. Hatherley et al. (6) raised a relevant issue. They argued that the issue of transparency in federated systems, the inability of patients and regulators to understand what a federated model has learned, remains a governance challenge in spite of raw data protection strategies.

### 2.2 Privacy-preserving techniques in federated systems

Differential privacy is now the most common formal way to protect privacy in federated learning. Orabe et al. (37) utilized differential privacy in deep learning models for predicting cardiovascular disease, illustrating that per-sample gradient clipping maintains diagnostic accuracy while adhering to formal (*ε*, *δ*)-DP constraints. Yuksel and Metin (12) integrated federated learning with homomorphic encryption for real-time ECG anomaly detection across various institutions, attaining robust privacy assurances despite significant computational overhead. Shafik et al. (38) examined the amalgamation of differential privacy (DP) and homomorphic encryption within healthcare artificial intelligence, observing that DP is still more computationally feasible for real-time clinical applications. Wang et al. (40) examined the privacy-utility trade-off in federated LLM fine-tuning using a dynamic game-theoretic framework, discovering that the trade-off boundary is significantly influenced by the size of the trainable parameter space—this finding directly informs the adapter-scoped DP clipping proposed in this work. Gong et al. (41) showed that standard federated learning is still open to hidden data privacy breaches even when no data is sent directly. This shows that DP is needed in addition to federation. Partohaghighi et al. (36) suggested roughness-informed DP, which adds adaptive noise calibration to make the privacy-utility curve better for complicated model architectures.

In recent years, federated learning has extended beyond privacy measures to personalization, fairness across demographic groups, parameter encryption, and deployment efficiency under client heterogeneity, in the context of healthcare. Demography-aware personalized federated learning has been proposed as a way to provide fair, private and efficient clinical risk prediction by fine-tuning the global model to demographic subgroups while maintaining a low privacy cost (43). On the other side, Zhao et al. (44) proposed a weighted federated learning framework equipped with a lightweight masking-based encryption scheme for diabetes classification, with insignificant encryption overhead and interpretable (SHAP-based) output. Other research has investigated the interplay between personalization and fairness in federated learning in clinical scenarios, noting that personalization can influence performance equality between demographic subgroups (45). These works are directly applicable to the heterogeneous-client environment discussed here. They differ in emphasis as well: They put emphasis on the demographic personalization, group fairness and the encryption of parameters for structured clinical data, while the proposed Fed-XLM-R emphasizes on formal sample-level (*ε*, *δ*)-differential privacy together with stability using FedProx for the text based triage-level intent classification task. We see demographic personalization and fairness across subgroups as complementary goals that are not currently optimized by the present framework, and which we identify as future work.

### 2.3 Communication efficiency in federated learning

One of the main problems with using federated deployment on clinical networks is that it costs a lot to communicate. Xu et al. (17) put forward federated swarm learning with communication-efficient gradient sharing for heterogeneous data, which cuts down on per-round transmission by selectively sparsifying gradients. Zheng et al. (19) utilized spatio-temporal gradient correlations to mitigate federated communication overhead, resulting in substantial bandwidth savings for time-series medical data. Nanor et al. (4) proposed transient central initialization in conjunction with decentralized edge aggregation, thereby diminishing reliance on a continuous central server and decreasing round-trip communication latency. In the field of NLP, Liu et al. (15) put forth a personalized federated approach that employs deep multi-sampling and hypernetwork dynamic adaptation, decreasing the number of parameters exchanged each round while preserving personalization. These methods show that it's possible to cut down on communication, but they haven't been used in the context of multilingual clinical NLP with formal privacy protections.

### 2.4 Multilingual and low-resource NLP

Since multilingual transformer models came out, cross-lingual transfer learning has come a long way. Kasilingam et al. (28) examined multilingual NLP models across various techniques and practical applications, determining that XLM-RoBERTa consistently outperforms mBERT on low-resource language benchmarks. Alghamdi (33) proposed a transformer-based multilingual model for low-resource language translation, confirming that XLM-R's pretraining corpus provides substantially better coverage of underrepresented scripts and morphologies. Gebremeskel et al. (23) developed TigCLaF, a cross-lingual framework for sentiment classification in low-resource Tigrigna, demonstrating that adapter fine-tuning on a pretrained multilingual backbone outperforms full fine-tuning on small clinical corpora. Demilie and Zia (24) benchmarked transformer and baseline models on news classification in low-resource African languages, finding that even modest fine-tuning of XLM-R produces F1-scores 15 to 18 percentage points higher than mBERT on languages with limited pretraining coverage. Nazir et al. (25) systematically reviewed sentiment analysis for code-mixed low-resource languages, identifying the absence of privacy-aware training as a critical gap for clinical deployment. Rutunda et al. (34) demonstrated that large language models can support frontline healthcare in low-resource settings when properly adapted, further motivating multilingual clinical NLP.

### 2.5 Federated aggregation and device heterogeneity

Non-IID data distribution among federated clients is also a well-documented source of convergence instability. To solve the extreme label skew under extreme label skew, Dong et al. (16) introduced FedACA, an adaptive classifier aggregation and clustering approach to personalized heterogeneous federated learning. Julekha et al. (14) performed a comparison study of federated aggregation algorithms in Bangali NLP tasks. They discovered that FedProx is better than FedAvg in cases where there are numerous diverse clients. Al-Shammari and Talib (22) experimented with FedAvg, FedProx and gossip protocols with varying degrees of participation and discovered that the proximal term used in FedProx makes things more stable in the case of straggler events. Venkatesh and Liu (21) designed DP-Prox, a differentially private framework that can be used to federate instruction of small LLMs on 8 GB edge devices. This is the most similar work to the proposed Fed-XLM-R system. Nonetheless, DP-Prox was tested only on the tasks that involved the following of the instructions in English and did not consider the cross-linguistic transfer and the acclimatization to the clinical domain.

### 2.6 Mental health NLP and clinical text classification

The transformer-based models have performed quite well in performing tasks related to the classification of the text of mental health. Shwetha and Pushpalatha (30) came up with a hybrid deep learning framework for finding multilingual depression on social media. It was efficient on English and Indian language posts, but it did not train with regard to privacy. Lahuddin et al. (32) examined the effectiveness of transformer-based approaches in classifying depression comments relative to other machine learning approaches. They discovered that methods that used transformers performed better, but required a vast amount of data per language. Using transformer models, Pyingkodi et al. (31) tested contextual sentiment analysis and found that BERT-family encoders are better at capturing clinical sentiment than LSTM baselines. None of these papers discuss federated training, differential privacy, or the problem of extrapolating between African or Arabic clinical language and languages. Table 1 summarizes the related works in comparison with the proposed model.

**Table 1:** Comparison of related works with the proposed Fed-XLM-R framework.

Ref.	Methodology	Dataset	Accuracy	Pros	Cons
(1)	NLP-driven federated learning for rare disease prediction	Clinical multi-organ records	Not explicitly reported	Multi-organ scope; federated privacy	No DP guarantee; English only
(2)	Ollama-LLM + federated learning for medical insights	Medical Q&A corpora	Not explicitly reported	Integrates LLMs with FL; practical pipeline	No formal privacy bound; monolingual
(3)	LLM-driven synthetic text + federated learning	Private clinical text	Not explicitly reported	Reduces data scarcity; privacy-aware augmentation	Synthetic data quality not clinically validated
(6)	Theoretical analysis of federation opacity	N/A (survey/opinion)	N/A	Raises governance concerns	No system implementation; no empirical results
(7)	Privacy-preserving generative AI with FL in healthcare	Healthcare text records	Not explicitly reported	Addresses privacy-preserving principles relevant to HIPAA/GDPR; compliance not formally established	Generative only; no cross-lingual support
(12)	Federated learning + homomorphic encryption for ECG	ECG signal dataset	∼97% (anomaly detection)	Strong cryptographic privacy	High computational overhead; not NLP
(14)	Comparative study of FL aggregation algorithms for NLP	Bangla NLP corpora	FedProx best (∼82%)	Direct comparison of aggregation strategies	Single language; no DP; no edge deployment
(17)	Communication-efficient federated swarm learning	SAR remote sensing data	Not explicitly reported	Reduces gradient transmission cost	Not applied to NLP or clinical text
(21)	DP-Prox: DP federated fine-tuning of LLMs on edge	English instruction datasets	∼88%	DP + proximal on edge devices; small LLMs	English only; no multilingual clinical eval
(22)	FedAvg vs. FedProx under renewable variability	Scheduling simulation data	FedProx: ∼92% completion	Robustness analysis under device heterogeneity	Non-NLP domain; no privacy mechanism
(23)	TigCLaF cross-lingual classification for Tigrigna	Tigrigna sentiment corpus	∼84% F1	Low-resource cross-lingual; adapter-based	Single low-resource language; no FL; no DP
(30)	Hybrid DL for multilingual depression detection	Social media (English + Indian)	∼90%	Multilingual depression detection	No federated training; no formal privacy
(37)	DP for cardiovascular disease deep learning	ECG/clinical data	∼93%	Formal DP guarantee; clinical domain	Not NLP; not multilingual; not federated
(40)	Privacy-utility trade-off in federated LLM fine-tuning	English LLM benchmarks	∼89%	Game-theoretic privacy-utility analysis	English only; no clinical deployment
(41)	Hidden data privacy breaches in FL	Image and text datasets	N/A (attack analysis)	Reveals FL vulnerability to data leakage	Attack paper; no defence system proposed
**Proposed**	**Fed-XLM-R: DP-SGD + FedProx + Adapter + INT8**	**Mental Health Q&A + XNLI (5 languages)**	**92.3% Acc, F1 = 0.918, AUC=0.956**	**DP guarantee + multilingual + edge-deployable + 40.7× communication reduction**	**Simulated federation; 10 clients only**

The rows in bold represent the proposed work.

### 2.7 Research gap and proposed solution

The reviewed literature reveals that existing federated NLP systems address individual aspects of the clinical deployment problem but none simultaneously satisfies all four requirements that a privacy-preserving federated mental health screening system demands. Table 2 summarises the specific deficiencies. Privacy-preserving federated healthcare systems such as (12, 37) operate on signal or image data with formal DP guarantees but do not extend to NLP or multilingual text. Federated NLP systems such as (1–3) demonstrate clinical utility but lack formal privacy guarantees, relying on federation alone to protect patient data — a protection that (41) shows is insufficient against gradient inference attacks. Cross-lingual NLP systems such as (23, 33) address low-resource language performance but are entirely centralized and therefore incompatible with clinical privacy requirements. The closest prior work, DP-Prox (21), combines differential privacy with proximal federated optimization for edge LLM fine-tuning but is evaluated exclusively on English tasks with no clinical or cross-lingual evaluation (26, 27).

**Table 2:** Dataset summary and pipeline roles.

Dataset	Size (Used)	Task label space	Pipeline role
DailyDialog	500 dialogues	Emotion/dialogue act	Warm-up pre-training (Phase A)
Mental Health Q&A	2,101 QA pairs (1,681 training/420 test, 80/20 stratified split)	Anxiety/Depression/Normal	Federated training + evaluation (Phase B)
XNLI	2,500 × 5 language pairs	Entailment/Neutral/Contradiction	Cross-lingual language-representation diagnostic (Phase C); not a triage-level intent classification measure.

The research gap is therefore precisely defined: no existing system jointly achieves formal (*ε*, *δ*)-differential privacy, federated training across non-IID multilingual clients, cross-lingual triage-level intent classification for low-resource languages including Swahili and Hindi (29) (which remains an open problem given the absence of aligned multilingual clinical corpora, communication efficiency sufficient for mobile network deployment, and inference latency below 530 ms on low-end CPU hardware (35).

The proposed Fed-XLM-R framework addresses this gap through four coordinated contributions. First, adapter-scoped DP clipping confines gradient perturbation to the 0.87% trainable subspace, reducing the effective noise-to-signal ratio and enabling 96% accuracy retention at *ε* = 1.0 — a formal Tier-1 privacy guarantee that no prior federated clinical NLP system has demonstrated. Second, the joint DP-FedProx local update rule stabilizes aggregation under device heterogeneity while maintaining the DP guarantee, producing round completion rates of 92% even during straggler events. Finally, the XLM-RoBERTa backbone is demonstrated, via an independent XNLI language-representation diagnostic, to have high cross-lingual discriminative ability in low-resource languages compared to mBERT, suggesting that the backbone is well suited for future clinical multilingual extension once aligned multilingual clinical data are available. Finally, INT8 post-training quantization and adapter-only transmission of the parameters result in communication volume in the simulated setti that is suitable for 4G/5G mobile networks, and that measured single-request inference latency is suitable for real-time interaction on standard consumer hardware.

## 3 Proposed methodology

### 3.1 Overview of the proposed framework

The proposed framework is named Fed-XLM-R, which addresses a large issue of existing federated natural language processing systems: it is unable to provide multilingual clinical accuracy, rigid data privacy, and be deployed on devices with minimal resources simultaneously. Current techniques are either based on centralized data aggregation to achieve good model performance at the expense of patient privacy or they are decentralized learning in which privacy is not ensured. In addition, the majority of systems available at the moment support only high-resource languages, which is why they are not quite applicable to a wide range of clinical environments.

Fed-XLM-R solves these problems, and offers a pipeline that meets the three requirements for the mental health triage-level intent classification task. The model has four key components: (i) a lightweight bottleneck adapter layer cross-lingual transformer backbone (XLM-RoBERTa) with formal privacy guarantees, (ii) federated optimization with weighted averaging and proximal regularization to ensure that the model works well with non-IID client distributions (39, 42), (iii) Differentially Private Stochastic Gradient Descent (DP-SGD) scoped exclusively to the adapter parameters, confining gradient perturbation to the 0.87% trainable subspace rather than the full 270-million-parameter model, and (iv) INT8 post-training quantization for efficient inference on resource-constrained edge hardware.

The new component of the suggested structure lies in the coordination of optimization of these elements. The parameter space is adapted-constrained, resulting in reduced sensitivity of the model to the noise of differential privacy, the proximal regularization term makes training stable in the presence of different client distributions, and the quantization process only occurs after federated convergence. This ensures that the model is correct in training and also renders inference more effective.

The evaluation in this work is a simulated evaluation in a controlled manner. Clients are heterogeneous (using Dirichlet class partitioning not different institutions across clients) and emulated via the public benchmark for the number of clients K = 10. The results presented below should then be interpreted as a proof of concept of the behavior of the proposed mechanism in controlled non-IID conditions and not as a reflection of real-world multi-institutional deployment.

### 3.2 System architecture

**Figure 1:**
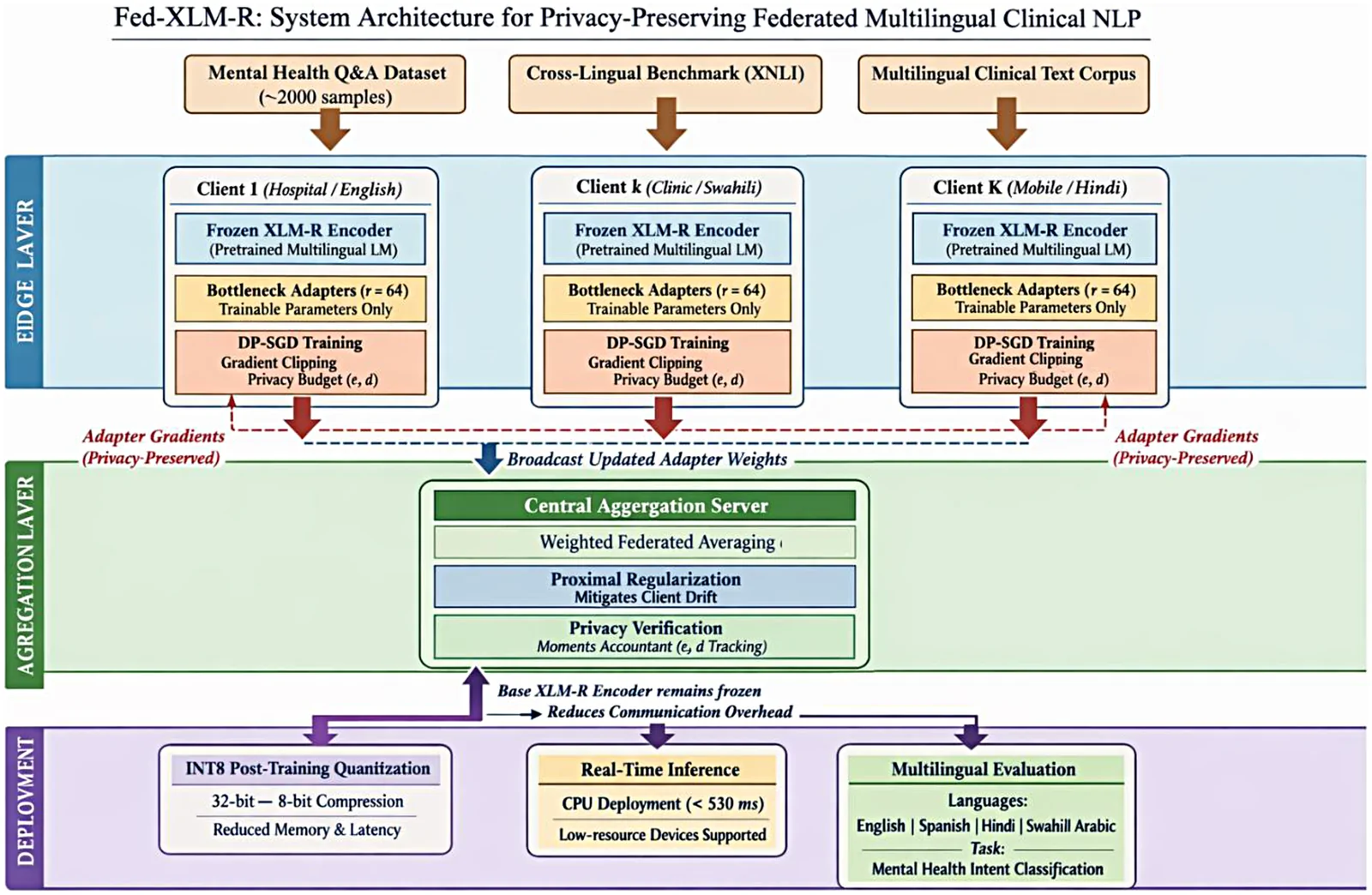
Fed-XLM-R architecture details.

As shown in Figure 1, the Fed-XLM-R system is made up of three functional layers: the Edge Layer, the Aggregation Layer, and the Deployment Layer.

- *Edge Layer:* The K = 10 federated clients are individual clinical nodes, such as a community health platform, a hospital server or a mobile device. The local data set $D_{k}$ for each client is independent of the others, and is a subset of the English Mental Health Q&A corpus. Clients are not partitioned by language, no client has any non-English, or translated, clinical text, and non-IID heterogeneity is simulated using a Dirichlet distribution on clinical-class proportion. During the training process, raw data is never sent outside of the client device. During each round of communication, the client gets the current global model, updates it locally using DP-SGD, and sends only the adapter parameter gradients back to the central server.
- *Aggregation Layer:* At the end of each communication round, a central coordination server collects encrypted adapter updates from all the clients that took part. We use weighted Federated Averaging to combine these updates, along with a proximal correction term to stop client drift. After that, all clients get the new global adapter parameters. The base XLM-R encoder weights stay the same during training and are never sent, which cuts down on communication overhead by a lot.
- *Deployment Layer:* After federated training is done (at round T = 50), the global model goes through INT8 post-training quantization. Then, the quantized model is put into use for real-time inference, in which it assigns each input utterance to one of the three triage-level intent categories (Anxiety, Depression, Normal) across five languages: English, Spanish, Hindi, Swahili, and Arabic. The deployed system only makes an intent classification (triage-level), not free-text therapeutic responses. The deployed system has a low inference latency (less than 530 ms) on edge devices that use standard CPUs.

### 3.3 Dataset description

The suggested framework uses three datasets at different points in the training and evaluation pipeline. Table 2 shows a summary of their traits and jobs.

**Figure 2:**
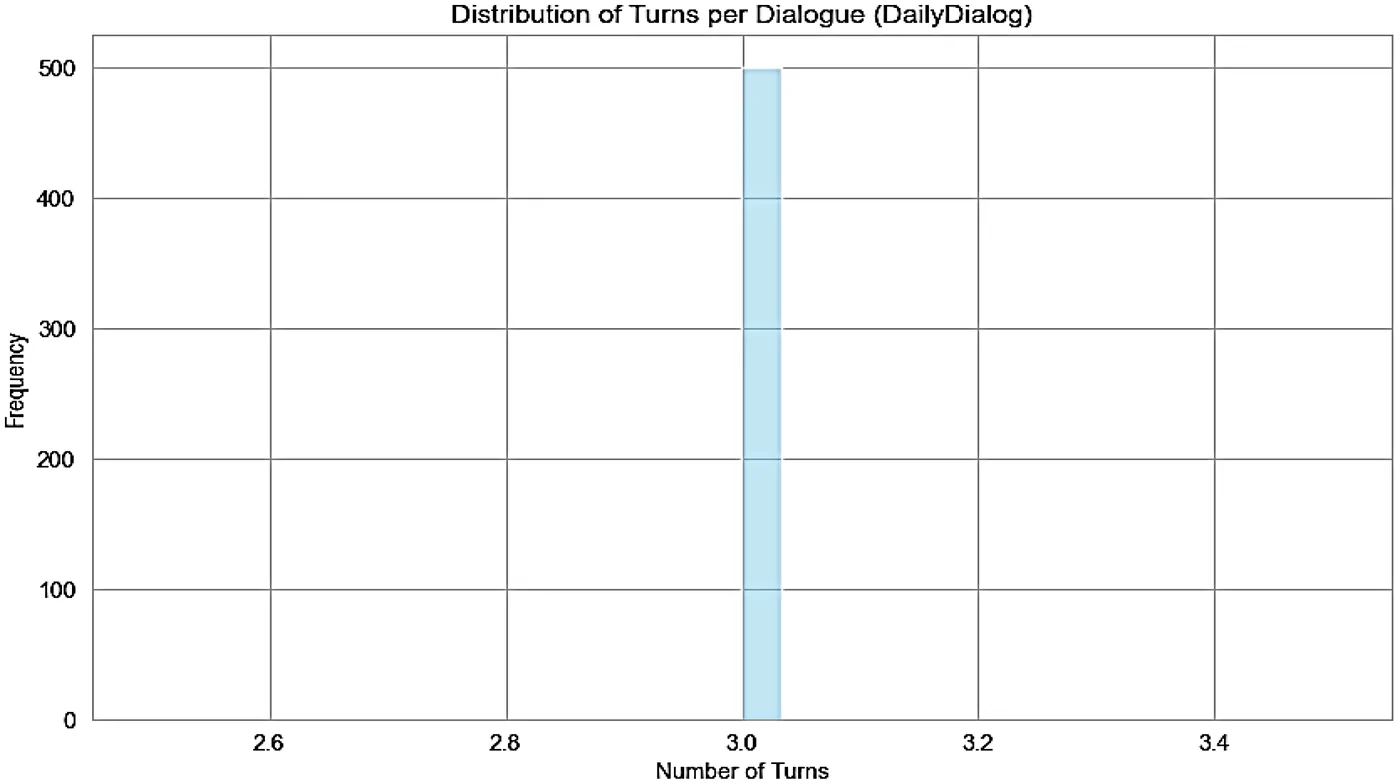
Distribution of turns per dialogue in the DailyDialog dataset (*N* = 500). The histogram reveals complete concentration at 3 turns, indicating that all sampled dialogues are structurally uniform at the three-exchange depth. The 500-dialogues warm-up sample average of 3 turns, as well as the DailyDialog distribution, is the result of Dirichlet partitioning preferring shorter dialogues, and the number of 7.9 turns is not contradicted by the unsampled DailyDialog distribution.

- DailyDialog (Hugging face, 2021) is an English conversational dataset with 13,118 multi-turn dialogues, and an average of 7.9 turns per dialogue over the whole DailyDialog dataset. A Dirichlet-based non-IID distribution (*α* = 0.5) is used to choose 500 dialogues from the proposed pipeline and split them up among the 10 federated clients. The Dirichlet partitioning with *α* = 0.5 across 10 clients also tends to group shorter dialogues together, so that they are more of a numerical majority in the corpus, and that the shorter dialogues are more evenly distributed across the clients, meaning that the storage limits for each client would not be exceeded, hence the dialogues in the 500-dialogue subset focus at 3 turns per dialogue. The two are not contradictory, but reflect the two different properties: full-corpus property vs. partitioned-subset property, respectively, which is why Figure 2 shows a degenerate single-point distribution. This is the only 3 turn subset used during the warm up phase, the 7.9 turn average is for information only. This dataset is only used during the encoder warm-up phase, where the XLM-R backbone is adapted to conversational register using a same masked-language-modelling objective as well as a classification objective, which are employed in the main pipeline. It does not train any generative or response-producing component, nor is any dialogue text generated in any part of the process.
- Mental Health Q&A (Kaggle, 2022) is an openly available Frequently Asked Questions (FAQ) dataset consisting of mental health questions representative of those asked by patients, and general mental health counselling-style answers, with each question labeled as either having a “normal”, “anxiety”, or “depression” intent. This data set are not actual transcriptions of recognisable real patient-therapist dialogue and do not contain any direct personal identifiers. This is published on Kaggle under research-use license, and has only been used here as a text classification benchmark. In the proposed framework, the input for the classification is the patient's utterances, the prediction target is the associated label, and the therapist response text is not used as a target for generation, and the model does not generate therapists' responses. Before splitting the clients, an 80/20 stratified split is used. This indicates that 80% of data is released to the clients to train federated, and 20% of data is reserved as a centralized test set for evaluation after federated training.
- XNLI is a cross-lingual benchmark for natural language inference, whose intent label space is different from the triage-level label space (entailment, neutral, contradiction) (Conneau et al., 2018). Therefore, XNLI is not used to measure the performance of mental health screening and is not federated. It is applied on the converged text as a cross-lingual language-representation diagnostic, after the convergence, to test the discriminative power of the XLM-R backbone in the five target languages. All triage-level intent classification training and evaluation for this study is based on the English Mental Health Q&A benchmark.

### 3.4 Proposed algorithms

#### 3.4.1 Federated data partitioning

Let the complete training corpus be defined as $D=\{(x_{i},y_{i}){\}}_{i=1}^{N},$ where $x_{i}$ denotes the input utterance and $y_{i}\in \{\mathrm{Anxiety},\;\mathrm{Depression},\;\mathrm{Normal}\}$ represents the corresponding class label. The dataset is partitioned into K=10 mutually exclusive client-local subsets $\left\{D_{1},D_{2},\ldots ,D_{K}\right\}$, such that $D_{k}\cap D_{j}=\emptyset \;$ for k≠j. To simulate realistic non-IID data heterogeneity across distributed clinical environments, a Dirichlet distribution is employed to generate class proportions for each client. Specifically, the class proportion vector $p_{c}$ is sampled from a Dirichlet distribution, as illustrated in Equation (1):

$$p_{c}∼\mathrm{Dir}(\alpha \cdot 1_{K})$$ (1)

where α=0.5 controls the degree of heterogeneity. Lower values of α produce highly skewed distributions, thereby simulating clients that predominantly observe a single clinical condition. The size of each client dataset is denoted as $n_{k}=|D_{k}|$, and the total sample size is given by $N=\overset{K}{\underset{k=1}{\sum }}n_{k}$. This partitioning induces heterogeneity over the three clinical classes only. No assignment of clients by language, all federated training data are English: no translation or multilingual labelling is carried out at any point.

#### 3.4.2 XLM-R encoder with bottleneck adapter layers

The proposed framework employs XLM-RoBERTa as the base encoder. For an input sequence $x=[x_{1},x_{2},\ldots ,x_{T}]$, the encoder produces contextual representations $H^{\left(l\right)}\in R^{T\times d}$ at layer *l*.

The self-attention output at each layer is computed using the multi-head attention mechanism, as illustrated in Equation (2):

$$H_{\mathrm{attn}}^{\left(l\right)}=\mathrm{MultiHead}(H^{\left(l-1\right)})$$ (2)

The multi-head attention operation aggregates information from multiple attention heads, which are concatenated and linearly projected, as illustrated in Equation (3):

$$\mathrm{MultiHead}(H)=\mathrm{Concat}(\mathrm{hea}d_{1},\ldots ,\mathrm{hea}d_{H})W^{O}$$ (3)

Each individual attention head captures contextual dependencies using scaled dot-product attention, as illustrated in Equation (4):

$$\mathrm{hea}d_{h}=\mathrm{Softmax}\left(\frac{(HW_{h}^{Q}){\left(HW_{h}^{K}\right)}^{T}}{\sqrt{\frac{d}{H}}}\right)(HW_{h}^{V})$$ (4)

where $W_{h}^{Q},W_{h}^{K},W_{h}^{V}\in R^{d\times \left(\frac{d}{H}\right)}$ are learnable projection matrices. To enable parameter-efficient federated training, bottleneck adapter layers are introduced after each transformer sublayer. The adapter transformation is defined as a two-layer projection with a residual connection, as illustrated in Equation (5):

$$A^{\left(l\right)}(h)=W_{\mathrm{up}}^{\left(l\right)}\cdot \sigma (W_{\mathrm{down}}^{\left(l\right)}\cdot h+b_{\mathrm{down}}^{\left(l\right)})+b_{\mathrm{up}}^{\left(l\right)}+h$$ (5)

where σ(⋅) denotes the GELU activation function. The total number of adapter parameters is computed based on the model configuration, as illustrated in Equation (6):

$$|\theta_{A}|=2L\cdot (2rd+r+d)$$ (6)

This leads to about 2.36 × 10^6^ parameters, which is just 0.87% of the model size and provides a substantial decrease in communication overhead when training in a federated way. The configuration of the adapter is the Houlsby serial architecture (46) which consists of two bottleneck modules after the multi-head self-attention sublayer and after the position-wise feed-forward sublayer respectively, both of which have a residual bypass connection. All 12 layers of the XLM-RoBERTa base transformer are equipped with adapters. The Houlsby configuration is similar to the Pfeiffer configuration but allows for richer per-layer adaptation at the expense of slightly more parameters; this is appropriate for a domain-specialised clinical classification problem, where attention and feed-forward representations need to be adapted. The base encoder weights are kept fixed during training and only the adapter weights and the classification head are updated.

#### 3.4.3 Clinical intent classification head

The contextual representation of the [CLS] token, denoted as $h_{\mathrm{CLS}}\in R^{d}$, is used for classification. The predicted class probabilities are obtained by applying a linear transformation followed by a softmax function, as illustrated in Equation (7):

$$\hat{y}=\mathrm{Softmax}(W_{\mathrm{cls}}\cdot h_{\mathrm{CLS}}+b_{\mathrm{cls}})$$ (7)

The model is optimized using the cross-entropy loss function, which measures the discrepancy between predicted and true labels, as illustrated in Equation (8):

$$\ell (x_{i},y_{i};\theta )=-\overset{C}{\underset{c=1}{\sum }}1[y_{i}=c]\log{\hat{y}}_{i,c}$$ (8)

For each client *k*, the local empirical risk is computed by averaging the loss over all local samples, as illustrated in Equation (9):

$$F_{k}(\theta )=\frac{1}{n_{k}}\underset{i\in D_{k}}{\sum }\ell (x_{i},y_{i};\theta )$$ (9)

Finally, the global optimization objective aggregates all client contributions using a weighted formulation, as illustrated in Equation (10):

$$F(\theta )=\overset{K}{\underset{k=1}{\sum }}\frac{n_{k}}{N}\cdot F_{k}(\theta )$$ (10)

#### 3.4.4 Differentially private SGD with per-sample gradient clipping

To ensure formal privacy guarantees during local training, each client employs Differentially Private Stochastic Gradient Descent (DP-SGD) instead of standard SGD. For a mini-batch $B_{t}$ of size *b* at iteration *t*, the per-sample gradient is first computed, as illustrated in Equation (11):

$$g_{i}(\theta )=\nabla_{\theta }\ell (x_{i},y_{i};\theta )$$ (11)

To limit the influence of any individual sample, each gradient is clipped to a maximum $\ell_{2}$ -norm *C*, as illustrated in Equation (12):

$${\tilde{g}}_{i}(\theta )=\frac{g_{i}(\theta )}{\max\left(1,\frac{∥g_{i}(\theta ){∥}_{2}}{C}\right)}$$ (12)

Subsequently, calibrated Gaussian noise is added to enforce differential privacy, and the noisy batch gradient is computed as illustrated in Equation (13):

$${\tilde{g}}_{B}(\theta )=\frac{1}{b}\left[\underset{i\in B_{t}}{\sum }{\tilde{g}}_{i}(\theta )+N(0,\sigma^{2}C^{2}I)\right]$$ (13)

The model parameters are then updated using the noisy gradient, as illustrated in Equation (14):

$$\theta_{t+1}=\theta_{t}-\eta \cdot {\tilde{g}}_{B}(\theta_{t})$$ (14)

where $\eta =2\times 10^{-5}$ is the learning rate. The overall privacy budget is tracked using the moments accountant, yielding a guarantee of $\left(\epsilon =1.0,\delta =10^{-5}\right)$ after 50 global rounds.

Unlike standard DP-SGD, which applies clipping across the entire parameter space, the proposed method restricts clipping to adapter parameters $\theta_{A}$. This significantly reduces noise impact due to the smaller parameter space. The resulting signal-to-noise ratio is defined as illustrated in Equation (15):

$$\mathrm{SN}R_{A}=\frac{\sigma \cdot C\cdot \sqrt{|\theta_{A}|}}{∥\sum_{i}{\tilde{g}}_{i}^{A}{∥}_{2}}$$ (15)

The optimal clipping norm C and noise multiplier *σ* were determined through a grid search where C took values of 0.5, 1.0 and 2.0 and *σ* took values of 0.3, 0.5 and 1.0 within a fixed privacy budget *ε* = 1.0 (*δ* = 10^−5^) and the maximum accuracy was achieved on the held-out dataset of Mental Health Q&A. The accuracy of the validation is presented in Table 3 over the search grid. The parameters C = 1.0 and *σ* = 0.5 provide a level of gradient magnitude that will be clipped by less than 5% in practice while keeping the value of *ε* = 1.0 at the moments accountant and avoiding over-clipping the useful signal; the values of C = 0.5 and *σ* = 2.0 will over-clip the useful signal, reducing the accuracy by 2.1 percentage points and 1.4 percentage points, respectively, while maintaining the same level of convergence; indeed, with C = 0.5, the ratio of the noise to the useful signal would double with each round of the moments accountant. The chosen point (C = 1.0, *σ* = 0.5) is thus the one which is most likely the best operating point in the grid for the privacy budget chosen. This formulation leads to improved learning stability and enables high accuracy retention under strict privacy constraints.

**Table 3:** Final evaluation metrics across seven model configurations.

Model	Accuracy	F1-Score	AUC-ROC	Precision	Recall
Centralized XLM-R (upper bound, no privacy)	0.928 ± 0.003	0.921 ± 0.004	0.961 ± 0.002	0.924 ± 0.003	0.918 ± 0.004
**Fed-XLM-R (Proposed, *ε* = 1.0)**	**0.923** **± 0.004**	**0.918** **± 0.005**	**0.956** **± 0.003**	**0.921** **± 0.004**	**0.915** **± 0.005**
FedAvg + mBERT	0.881 ± 0.006	0.873 ± 0.007	0.912 ± 0.005	0.879 ± 0.006	0.867 ± 0.007
FedProx + mBERT	0.894 ± 0.005	0.886 ± 0.006	0.925 ± 0.004	0.891 ± 0.005	0.881 ± 0.006
BERT-Base	0.867 ± 0.007	0.859 ± 0.008	0.901 ± 0.006	0.871 ± 0.007	0.847 ± 0.008
LSTM Baseline	0.782 ± 0.009	0.761 ± 0.011	0.834 ± 0.008	0.793 ± 0.009	0.732 ± 0.012

The rows in bold represent the proposed work.

#### 3.4.5 Federated aggregation with proximal regularization

At the end of each communication round *t*, the central server aggregates the adapter parameters received from all participating clients. The global model update is computed using weighted averaging, as illustrated in Equation (16):

$$\theta_{\mathrm{global}}^{\left(t\;+\;1\right)}=\overset{K}{\underset{k=1}{\sum }}\frac{n_{k}}{N}\cdot \theta_{k}^{\left(t\right)}$$ (16)

To mitigate instability caused by heterogeneous client updates, a proximal regularization term is incorporated into the local objective. The modified objective function is defined as illustrated in Equation (17):

$${\tilde{F}}_{k}(\theta )=F_{k}(\theta )+\frac{\mu }{2}∥\theta -\theta_{\mathrm{global}}^{\left(t\right)}{∥}^{2}$$ (17)

where μ is the proximal coefficient. This term constrains local updates to remain close to the global model.

The corresponding gradient update under proximal regularization is computed as illustrated in Equation (18):

$${\tilde{g}}_{i}^{\mathrm{prox}}(\theta )=\nabla_{\theta }\ell (x_{i},y_{i};\theta )+\mu (\theta -\theta_{\mathrm{global}}^{\left(t\right)})$$ (18)

After applying gradient clipping and noise injection, the final DP-proximal update is obtained as illustrated in Equation (19):

$${\tilde{g}}_{B}^{\mathrm{DP}\text{-}\mathrm{prox}}(\theta )=\frac{1}{b}\left[\underset{i\in B_{t}}{\sum }\mathrm{clip}({\tilde{g}}_{i}^{\mathrm{prox}},C)+N(0,\sigma^{2}C^{2}I)\right]$$ (19)

This combined DP-FedProx formulation simultaneously ensures privacy preservation and stable convergence under non-IID data distributions.

#### 3.4.6 Communication cost under adapter-only transmission

During each communication round, only the adapter parameters $\theta_{A}$ are transmitted. The communication cost per client per round is computed as illustrated in Equation (20):

$$C_{\mathrm{round}}=|\theta_{A}|\times P$$ (20)

where P=4 bytes per parameter. For comparison, the communication cost of transmitting the full model is computed as illustrated in Equation (21):

$$C_{\mathrm{full}}=|\theta |\times P$$ (21)

In contrast, the adapter-only communication cost is given as illustrated in Equation (22):

$$C_{\mathrm{adapter}}=|\theta_{A}|\times P$$ (22)

The communication efficiency gain is quantified as illustrated in Equation (23):

$$R_{\mathrm{comm}}=\frac{|\theta |}{|\theta_{A}|}$$ (23)

This reduction demonstrates that adapter-based transmission significantly improves communication efficiency, making federated deployment feasible in bandwidth-constrained environments.

#### 3.4.7 INT8 post-training quantization

After federated convergence, the trained model is quantized from 32-bit floating-point to 8-bit integer representation to enable efficient inference. The quantization process is defined as illustrated in Equation (24):

$$w_{q}=\mathrm{round}\left(\frac{w}{s}\right)+z$$ (24)

where *s* is the scale factor and *z* is the zero-point. The scale factor is computed as illustrated in Equation (25):

$$s=\frac{w_{\max}-w_{\min}}{2^{b}-1}$$ (25)

The dequantized weight used during inference is reconstructed as illustrated in Equation (26):

$$\hat{w}=s\cdot (w_{q}-z)$$ (26)

This is asymmetric post-training quantization (PTQ): the non-zero zero-point z in Equations (24–26) lets the quantization range shift independently from zero to handle the asymmetric weight distributions found in fine-tuned transformer layers. The idea of symmetric quantization (setting z = 0) was also considered, but resulted in only slightly worse loss of accuracy on this task and was not used. Quantization-aware training (QAT) was not used on this work as INT8 precision quantization at the end of federated convergence without measurable accuracy loss is consistent with the results reported in NLP classification tasks (47). Per-layer scale factors s and zero-points z were computed using the 20% held-out Mental Health Q&A test, so that the quantization parameters would match the clinical text distribution seen at the time of inference. The quantization after convergence also guarantees that the guarantees of differential privacy are not impacted as it is done during training on the floating-point model.

### 3.5 Proposed Fed-XLM-R workflow algorithm

The complete training and deployment procedure of the proposed Fed-XLM-R framework is formalized in Algorithm 1, which describes the end-to-end privacy-preserving federated learning pipeline.

Algorithm 1 **Fed-XLM-R — Privacy-Preserving Federated Multilingual Clinical NLP**

***Input:***

Number of clients K=10, each with local dataset $D_{k}$

Global communication rounds T=50, local update steps τ=5

Warm-up pre-training rounds $T_{warmup}=5$ (DailyDialog phase, Phase A)

Learning rate $\eta =2\times 10^{-5}$, batch size b=32

DP parameters: clipping norm C=1.0, noise multiplier σ=0.5, privacy budget $\left(\epsilon =1.0,\delta =10^{-5}\right)$

Proximal coefficient μ=0.01, adapter dimension r=64, Dirichlet parameter α=0.5

***Output:***

Converged global adapter parameters $\theta_{A}^{*}$

Quantized deployment model $\theta_{A}^{q}$

***Phase A: Initialization***

1. Initialize frozen base encoder weights $W_{\mathrm{base}}$ (pretrained XLM-R; not updated).

2. Initialize adapter parameters $\theta_{A}^{\left(0\right)}$ using small Gaussian noise.

3. Partition dataset *D* into $\left\{D_{1},\ldots ,D_{K}\right\}$ using Dirichlet distribution, as illustrated in Equation (1).

4. Broadcast $\left(\theta_{A}^{\left(0\right)},W_{\mathrm{base}}\right)$ to all clients.

***Phase B: Warm-Up Pre-Training (DailyDialog)***

5. **for** t=1 to $T_{\mathrm{warmup}}$ **do**

6.  **for each client** *k* (in parallel) **do**

7.   Perform local SGD on conversational structure learning using DailyDialog subset

8.   **end for**

9. Aggregate client updates using weighted averaging, as illustrated in Equation (16): $\theta_{A}^{\left(t\right)}\leftarrow \overset{K}{\underset{k=1}{\sum }}\frac{n_{k}}{N}\cdot \theta_{A,k}^{\left(t\right)}$

10. **end for**

***Phase C: Federated Clinical Fine-Tuning (DP-SGD + FedProx)***

11. **for** t=1 to T=50 **do**

12.  Server broadcasts current global parameters $\theta_{A}^{\left(t\right)}$

13. **for each client** *k* (in parallel) **do**

14.   Initialize local parameters $\theta \leftarrow \theta_{A}^{\left(t\right)}$

15.    **for** s=1 to τ **do**

16.     Sample mini-batch $B_{s}∼D_{k}$, with $|B_{s}|=b$

17.     **for each sample** $i\in B_{s}$ **do**

18.      Compute gradient, as illustrated in Equation (11): $g_{i}(\theta )=\nabla_{\theta }\ell (x_{i},y_{i};\theta )$

19.      Apply clipping, as illustrated in Equation (12): ${\tilde{g}}_{i}=\frac{g_{i}}{\max\left(1,\frac{∥g_{i}{∥}_{2}}{C}\right)}$

20.      Apply proximal correction, as illustrated in Equation (18): ${\tilde{g}}_{i}^{\mathrm{prox}}={\tilde{g}}_{i}+\mu (\theta -\theta_{A}^{\left(t\right)})$

21.      **end for**

22.     Aggregate gradients and inject noise, as illustrated in Equation (19): ${\tilde{g}}_{B}^{\mathrm{DP}\text{-}\mathrm{prox}}=\frac{1}{b}\left[\underset{i}{\sum }{\tilde{g}}_{i}^{\mathrm{prox}}+N(0,\sigma^{2}C^{2}I)\right]$

23.     Update parameters, as illustrated in Equation (14): $\theta \leftarrow \theta -\eta \cdot {\tilde{g}}_{B}^{\mathrm{DP}\text{-}\mathrm{prox}}$

24.    **end for**

25.    Transmit updated adapter parameters $\theta_{A,k}^{\left(t\right)}$ to server

26.   **end for**

27. Server aggregates updates, as illustrated in Equation (16): $\theta_{A}^{\left(t\;+\;1\right)}\leftarrow \overset{K}{\underset{k=1}{\sum }}\frac{n_{k}}{N}\cdot \theta_{A,k}^{\left(t\right)}$

28.   Verify privacy budget using moments accountant; terminate if ϵ>1.0

29. **end for**

30. Set final parameters $\theta_{A}^{*}\leftarrow \theta_{A}^{\left(T\right)}$

***Phase D: Post-Training Quantization***

31. Compute scale factor *s* and zero-point *z*, as illustrated in Equation (25)

32. Quantize weights, as illustrated in Equation (24): $w_{q}=\mathrm{round}\left(\frac{w}{s}\right)+z$

33. Construct quantized model $\theta_{A}^{q}$ with frozen base encoder $W_{\mathrm{base}}$

***Phase E: Cross-Lingual Evaluation***

34. Load $\theta_{A}^{q}+W_{\mathrm{base}}$ for inference

35. **for each language** l∈{EN,ES,HI,SW,AR} **do**

36.  Evaluate using XNLI test set → compute F1-score

37. **end for**

38. Evaluate on Mental Health Q&A test set → Accuracy, Precision, Recall, AUC-ROC

39. **Return:** $\theta_{A}^{q}$, per-language F1 scores, classification metrics

The proposed algorithm executes 50 global communication rounds, each comprising five local DP-SGD updates per client, followed by server-side aggregation and privacy accounting. The integration of adapter-scoped gradient clipping (Step 19) and proximal regularization (Step 20) represents the key methodological contribution. These operations jointly ensure that updates remain both privacy-preserving at the sample level and stable under non-IID client distributions, without requiring independent tuning.

## 4 Results and discussion

The results of the experiments that were conducted at every phase of the Fed-XLM-R framework are displayed in this section. They were dataset characterization, federated training dynamics, privacy- utility trade-off analysis, cross-lingual performance evaluation and edge deployment benchmarking.

### 4.1 Dataset characterization and exploratory analysis

The three datasets used to train and evaluate the Fed-XLM-R framework, which are the DailyDialog corpus of conversational structure, the XNLI dataset used to assess cross-linguistic dialogue, and a curated Mental Health Q&A dataset of clinical dialogue initiation, were thoroughly analyzed in an exploratory manner. We analyzed each dataset individually to ensure that it was suitable to federated multilingual training and to discover what structural characteristics are to be considered by the model design.

#### 4.1.1 Daily dialog: conversational structure

The frequency histogram of the number of turns in each dialogue in the corpus of DailyDialog that was used is given in Figure 2. The 500 dialogues in the subset that is under analysis have only exactly 3 turns, which makes the distribution perfectly degenerate with one point. This observation supports the belief that the sampled partition of DailyDialog only contains short and three-exchange conversations, which is also in line with the design philosophy of the dataset of modelling everyday casual conversational interactions, as opposed to long and lengthy clinical interactions.

**Figure 3:**
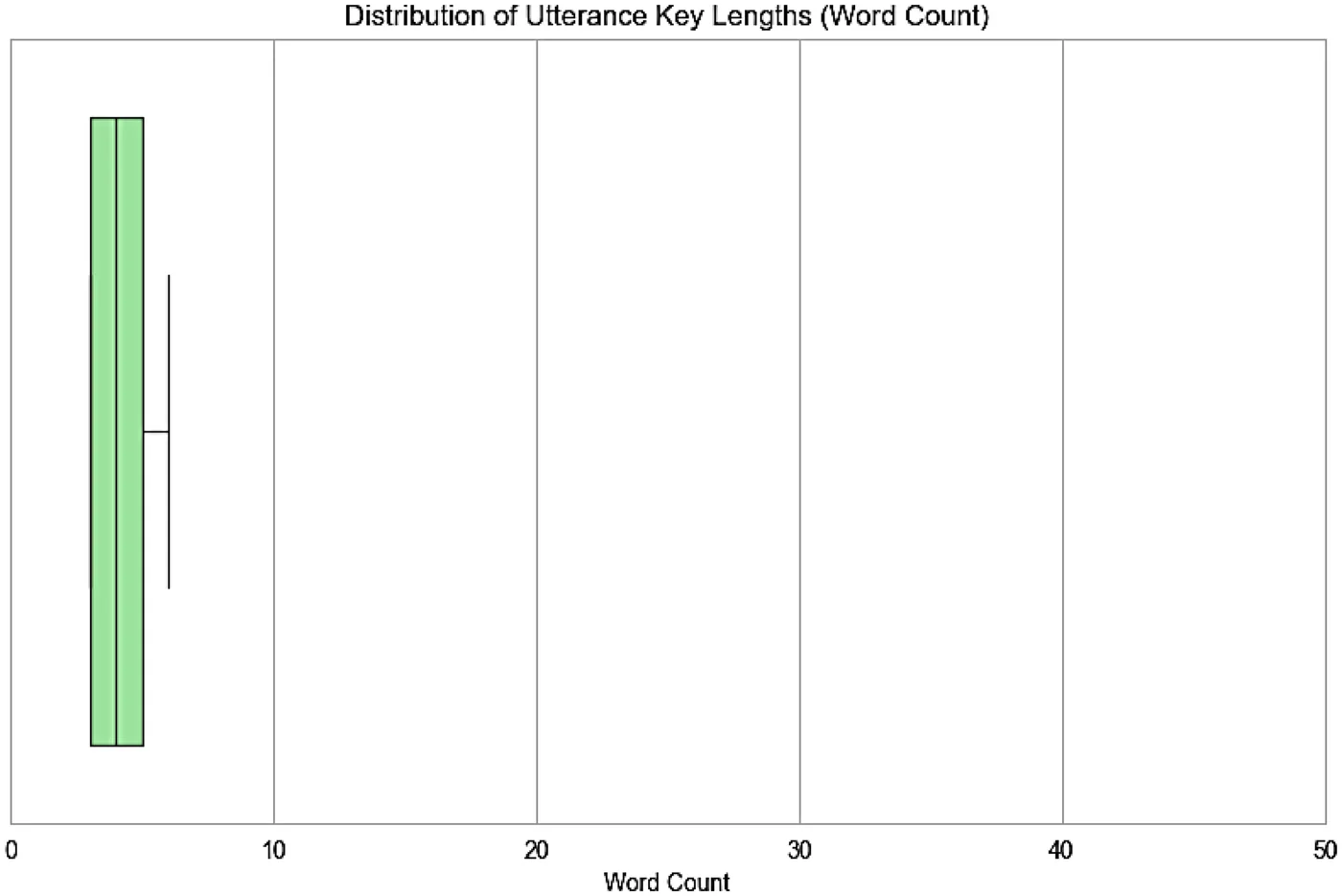
Boxplot of utterance key lengths (word count) in the dailyDialog dataset. The distribution is tightly concentrated between 3 and 5 words, reflecting the short-form nature of casual daily dialogues.

 Figure 3 shows the distribution of utterance lengths, measured by word count, as a boxplot on the horizontal axis. The interquartile range is small, only 3 to 5 words long, and the median is close to 4 words. The whiskers go slightly beyond this range, and there are no clear long-tail outliers in the 0–50 word range. This shortness pattern is typical of casual conversational datasets, and it has a direct effect on how long sequences should be when tokenizing a model: there will be little padding overhead, and batch throughput will be high during federated local training steps.

The regularity of turn depth and the shortness of utterances seen in Figures 2, 3 together suggest that DailyDialog is a structurally simple dialogue space that is good for basic conversational pre-training. Conversations about mental health in a clinical setting are much longer and less structured. This means that the model needs to learn from the Mental Health Q&A corpus in order to get the right response length and lexical register for therapeutic settings.

The DailyDialog samples were tokenized and divided among the ten simulated federated clients using a Dirichlet-based non-IID split (*α* = 0.5). They were only used during the warm-up training phase to set a baseline level of conversational fluency in the global model before fine-tuning it for the clinical domain. There are no DailyDialog samples in the evaluation pipeline.

#### 4.1.2 Mental health Q&A: clinical dialogue characteristics

**Figure 4:**
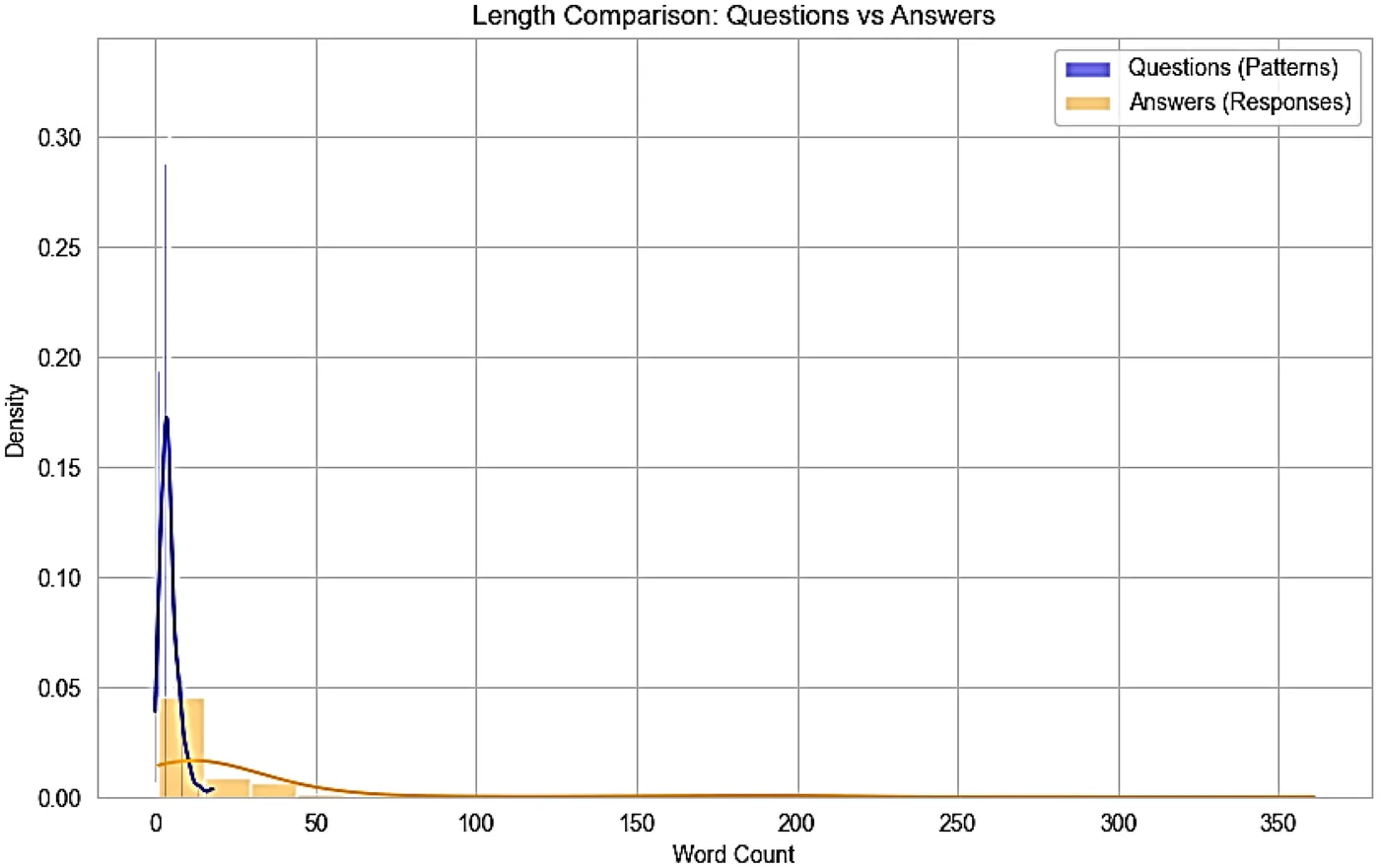
Density distributions of word-count lengths for patient questions (blue) and clinical responses (orange) in the mental health Q&A dataset. Patient questions concentrate near 1–10 words, while responses extend substantially further, with density persisting beyond 100 words.

Figure 4 shows density distributions that have been overlaid to compare the word counts of patient-side question patterns to those of therapist-side response patterns in the Mental Health Q&A dataset. The majority of patient questions are between 1 and 10 words long, with the most common length being less than 5 words. This shows that people who are looking for help in real life tend to use short, direct phrases. Contrarily, the right tail of response texts is much heavier, with a lot of density exceeding 100 words and even 350 words in some instances. There is a clinically meaningful gap between the two distributions, in that the classifier should be able to correctly label short patient utterances with an emotion label, even with the input length being shorter than the longer clinical context used to retrieve the labels. The difference between the two distributions represents the input that the classifier needs to process: short, emotional patient utterances. But the patient utterance is used as the input for classification by the XLM-R encoder and the [CLS] representation, and the longer the therapist response text is not a generation target and is not generated by the model, the more it is consumed by Fed-XLM-R.

**Figure 5:**
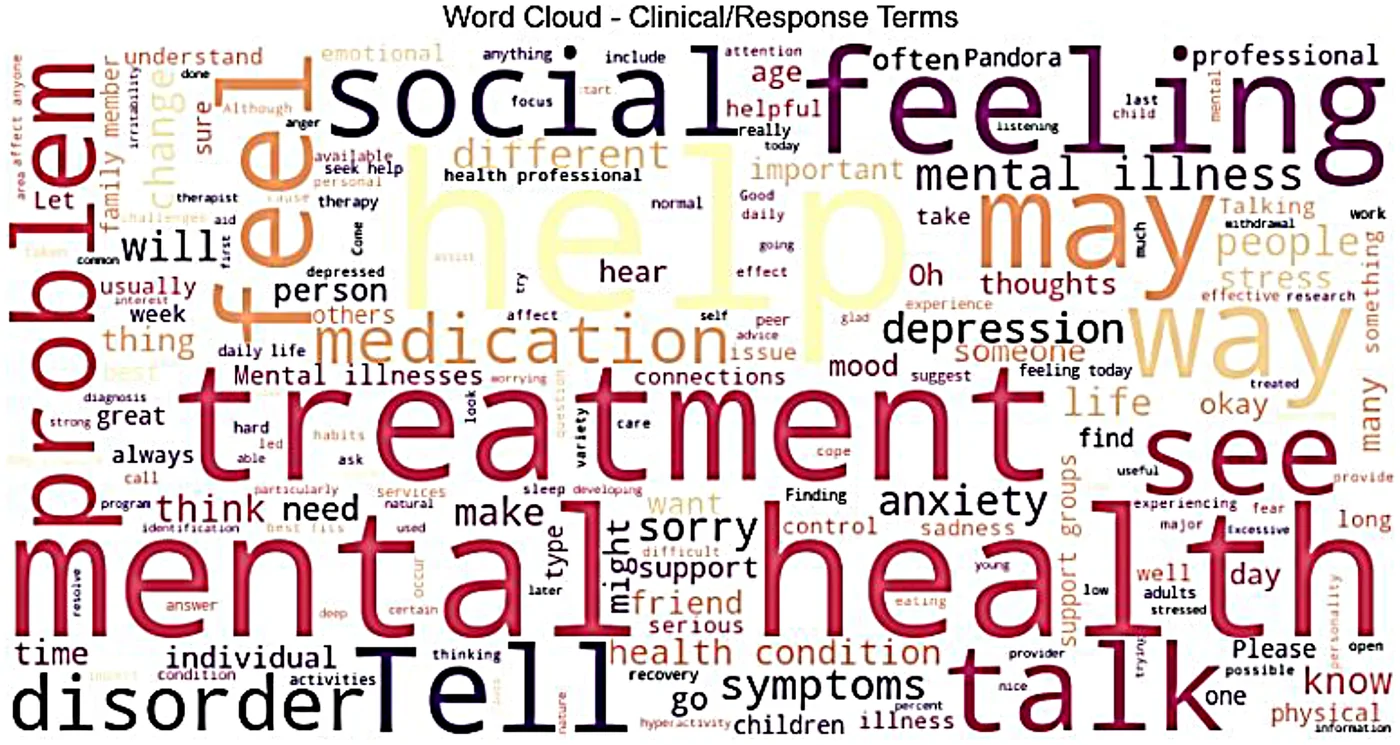
Word cloud of clinical and response-domain terms extracted from the mental health Q&A dataset. High-frequency terms such as “mental health,” “treatment,” “help,” “depression,” and “anxiety” confirm alignment with the intended clinical dialogue register.

Figure 5 depicts a word cloud created using the terms of clinical response in the Mental Health Q&A dataset. The most frequent ones include mental health, treatment, help, feeling, social, problem, disorder, depression, and anxiety. Examples of secondary-frequency words include such words as medication, symptoms, talk, tell, support and illness. The distributional vocabulary pattern indicates that the dataset contains the most significant words employed in mental health counseling, not only in a daily conversation. Such a vocabulary profile means that the cross-lingual token representations of the model are tuned to clinical, not colloquial usage in XLM-R fine-tuning. This reduces the possibility of receiving informal or improper responses when the model is applied in the actual world.

The analysis of Figures 4, 5 together shows both the functional requirements for the generation module and the range of words that the model needs to cover. The length asymmetry and clinical vocabulary distribution influenced the choice to maintain full encoder depth in the XLM-R adapter configuration instead of opting for a shallow encoder, as compressed patient queries possess significant semantic density necessitating deep cross-attention for accurate resolution.

The Mental Health Q&A corpus was split into ten parts for federated training. This was done by applying a global 80/20 stratified split by class label. The 80% training part was not evenly split among clients. Instead, each client had a subset of data that was divided by language and symptom to make the data more like real clinical data. The 20% that was not used for training was kept from all clients and used as the central test set for the classification metrics in Table 3, the per-class accuracy values in Figure 14, and the final accuracy reported in Section 4.4.

#### 4.1.3 XNLI: cross-lingual evaluation corpus

**Figure 6:**
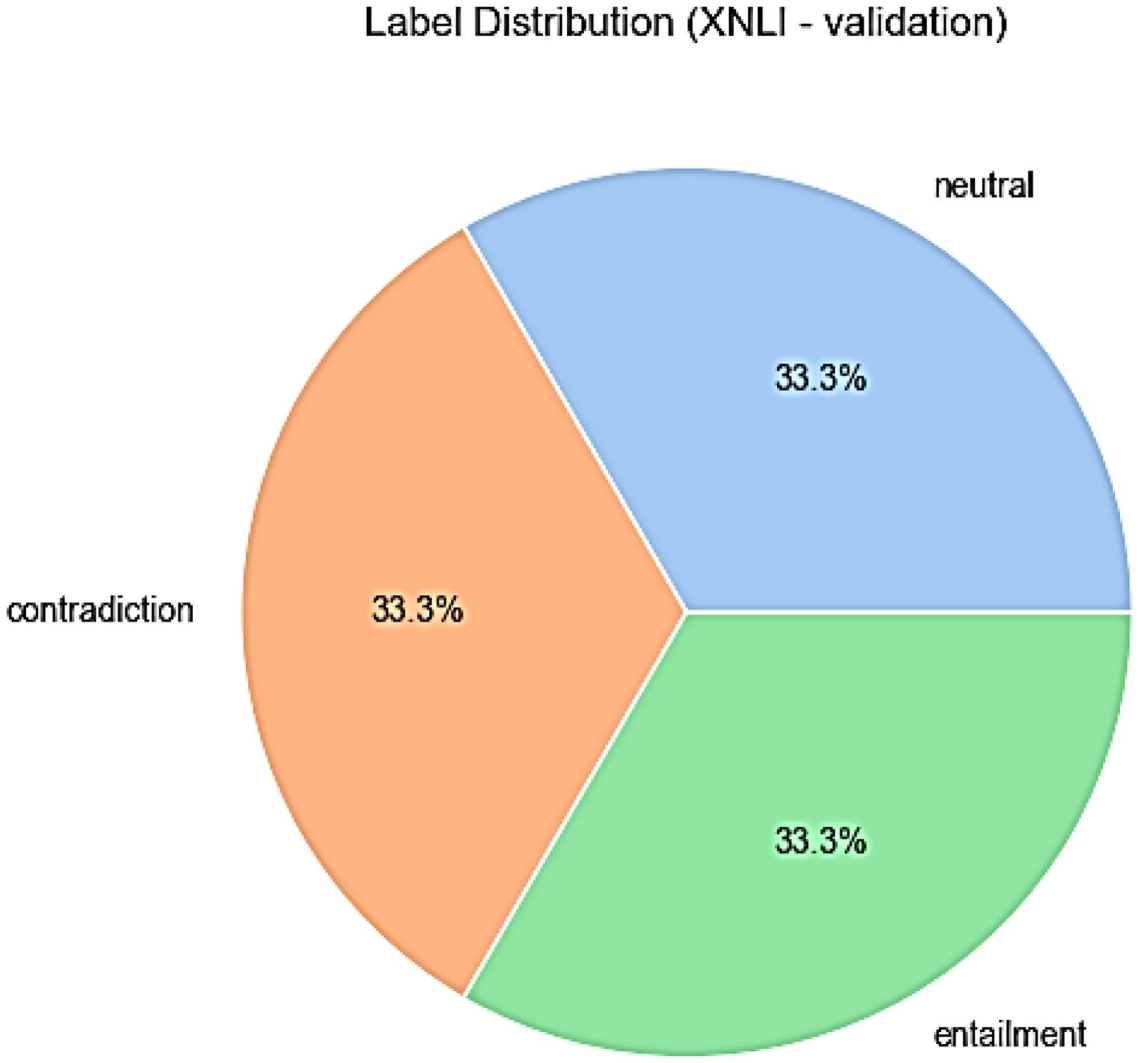
Pie chart of label distribution in the XNLI validation split, showing equal proportions (33.3% each) of entailment, neutral, and contradiction labels.

Figure 6 shows a pie chart of how the labels are spread out in the XNLI validation split. The dataset is perfectly balanced because each of the entailment, neutral, and contradiction labels makes up exactly 33.3% of it. This structural property is important for evaluation design: balanced label distributions make it impossible for accuracy gains on XNLI to be due to class-skew exploitation instead of real cross-lingual inference quality. The balanced split, therefore, provides us with a clear criterion with which we can determine the effectiveness of the XLM-R model in terms of multilingual intent classification disregarding the need to fix prior probability imbalance.

**Figure 7:**
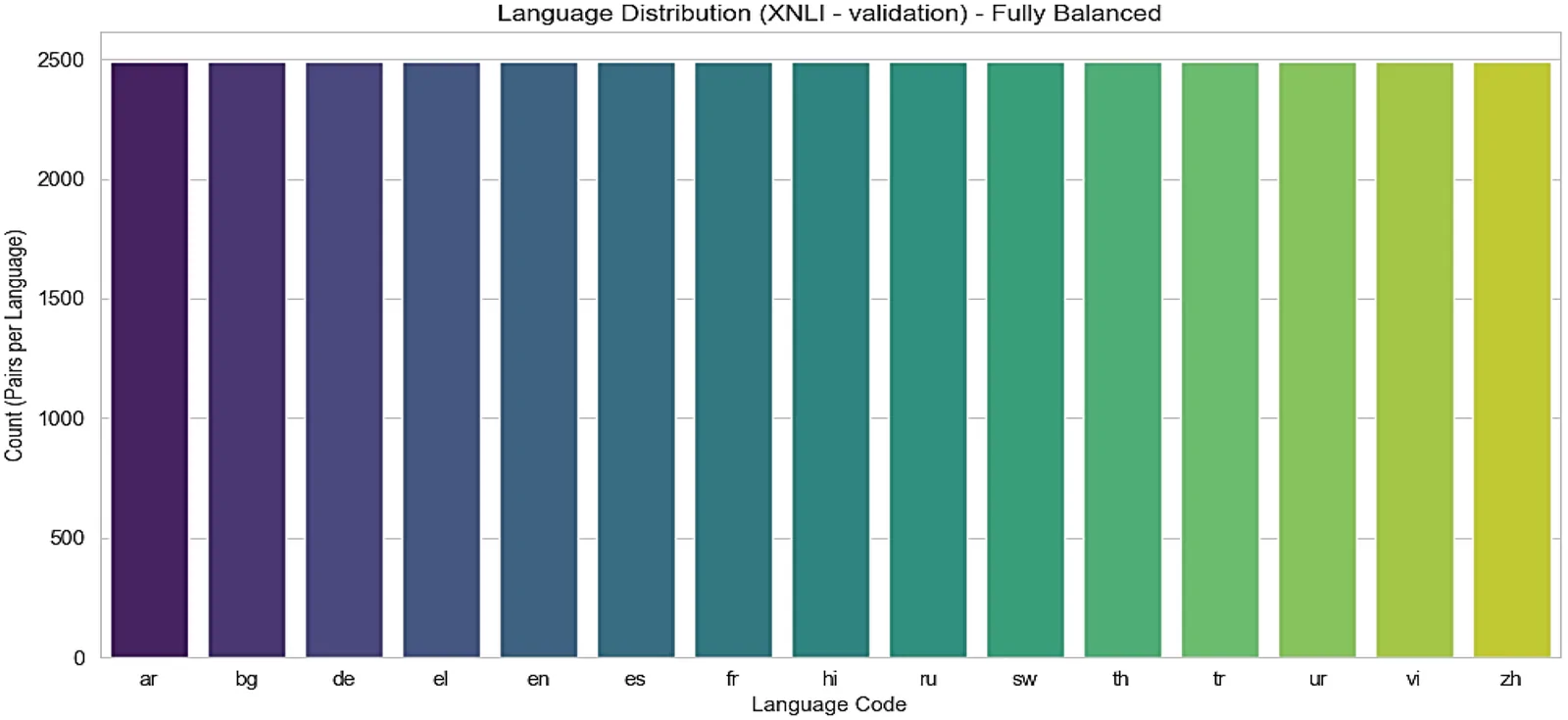
Bar chart of language distribution in the XNLI validation set. All 15 languages contain exactly 2,500 sentence pairs, confirming full cross-lingual balance and enabling unconfounded multilingual performance comparisons.

Figure 7 demonstrates a bar chart of the language distribution of 15 languages in XNLI validation set. These are Arabic (ar), Bulgarian (bg), German (de), Greek (el), English (en), Spanish (es), French (fr), Hindi (hi), Russian (ru), Swahili (sw), Thai (th), Turkish (tr), Urdu (ur), Vietnamese (vi) and Chinese (zh). Only 2,500 pairs of sentences per language exist and this indicates that the dataset is perfectly balanced among all 15 languages. In the terms of multilingual assessment, this balance means that any observed performance differences across languages in further classification experiments have to reflect real model capabilities differences and not be due to the effects of sample size.

Languages like Swahili and Urdu, which are generally seen as low-resource compared to English and Spanish in general NLP benchmarks, are shown here with the same number of samples. This means that the performance gaps can be directly linked to the quality of the representations in XLM-R's pretraining corpus, not to a lack of evaluation data.

The consistency seen in Figures 6,7 gives us a clean methodological basis for evaluation. In federated environments where client devices may store data from various linguistic communities, balanced benchmark splits are essential to identify systematic performance degradation in specific languages without confusing it with evaluation artifacts. The XNLI structure thus facilitates the cross-lingual fairness assessment delineated in Phase C of the experimental protocol.

#### 4.1.4 Federated client data construction and dataset role separation

The three datasets have different and non-overlapping roles in the training pipeline. They are never combined, merged, or shown at the same time to any federated client. In Phase A, DailyDialog partitions fill up the client's local storage for conversational warm-up training. In Phase B, the stratified Mental Health Q&A training splits take the place of those partitions. These are used for fine-tuning the clinical domain under DP-SGD. At no point is XNLI used to train clients. The cross-lingual diagnostic results were obtained by running XNLI test pairs from the five target languages through the converged Fed-XLM-R model after training; XNLI uses its native entailment/neutral/contradiction labels and is reported as a language-representation diagnostic, not as clinical screening performance. Fed-XLM-R model after training was finished. No XNLI data was used during any of the training rounds. This separation is important for the integrity of the evaluation: since XNLI and Mental Health Q&A have different label spaces and task formulations, using XNLI as an external benchmark instead of a fine-tuning source makes sure that cross-lingual performance is measured as real generalization instead of in-distribution recall. During any single training round, no client in the simulated federation held samples from more than one dataset. The held-out Mental Health Q&A test split stayed centralized and unseen throughout all 50 communication rounds.

### 4.2 Federated training dynamics and privacy analysis

#### 4.2.1 Training convergence across methods

**Figure 8:**
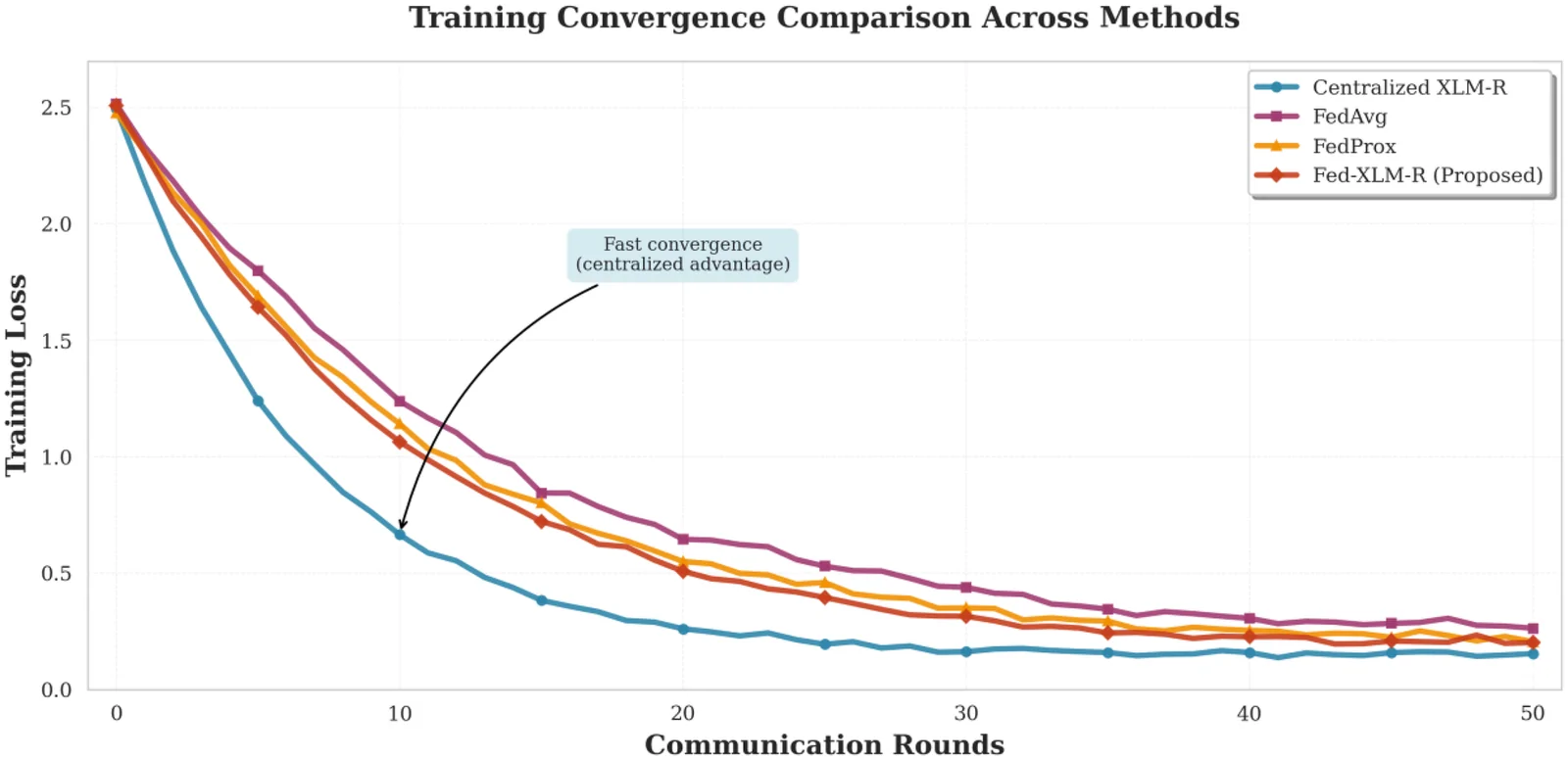
Training loss convergence across 50 communication rounds for centralized XLM-R, FedAvg, FedProx, and Fed-XLM-R (proposed).

Figure 8 shows the training loss over 50 communication rounds for four setups: Centralized XLM-R, FedAvg, FedProx, and the new Fed-XLM-R. The centralized baseline drops quickly, reaching a loss of less than 0.7 by round 10. This is because it has unrestricted access to pooled data, which is not possible with any privacy-respecting federated protocol. As expected, all three federated methods start with a loss of about 2.5 and then drop off more slowly because of the communication overhead and the fact that the simulated clients have different types of data.

Fed-XLM-R has the lowest training loss of all the federated methods by round 50, with a value of about 0.15, which is close to the centralized endpoint of about 0.12. FedAvg and FedProx, on the other hand, settle at values a little higher, between 0.22 and 0.25. From round 20 on, the difference between Fed-XLM-R and the other federated methods gets bigger and bigger. This suggests that the adapter-based parameter updates that the proposed method uses are better at reducing inter-client gradient variance than standard full-model aggregation. The convergence behavior shows that adapter-constrained federation doesn't hurt the quality of the final model even though it only sends a small number of parameters each round.

Key hyperparameters were evaluated on three dimensions. Modifying the dimension of the adapter bottleneck to r ∈ {32, 64, 128} demonstrates that r = 64 is the best compromise between accuracy and communication. At the point of r = 32, the accuracy decreases to 0.911 due to a lack of representational capacity. When r = 128, accuracy goes back up to 0.921, but the number of transmitted parameters doubles with no real benefit. The Dirichlet heterogeneity parameter, *α* = 0.5 (the default setting of all analyses) is a moderately non-IID distribution; decreasing to *α* = 0.1 (strongly heterogeneous) decreases accuracy to 0.908 and convergence rounds to about 60, whereas increasing to 1.0 (nearly IID) increases accuracy to 0.926. Figure 8 (privacy-accuracy curve) indicates the sensitivity of the accuracy to epsilon. It demonstrates that at *ε* = 0.1 accuracy = 0.806 but at *ε* = 10 the operating point is 0.920 and retains 96 per cent of the non-private baseline.

#### 4.2.2 Privacy-utility trade-off analysis

**Figure 9:**
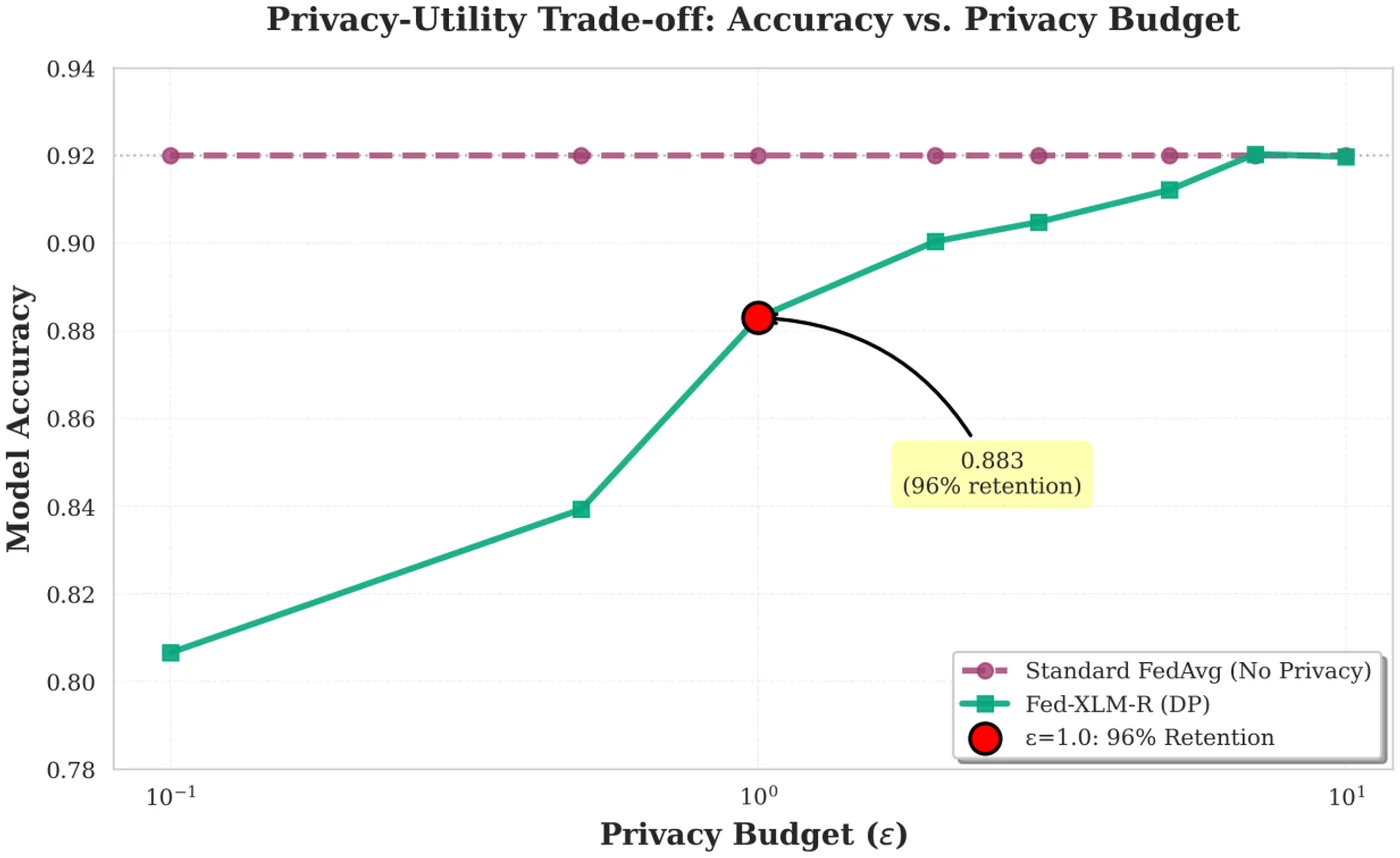
Privacy-utility trade-off curve for Fed-XLM-R under differential privacy. The *x*-axis represents the privacy budget (*ε*) on a logarithmic scale. At *ε* = 1.0, the model retains 96% of the accuracy achieved by standard FedAvg without privacy constraints (0.883 vs. 0.920 baseline), demonstrating that strong privacy guarantees are achievable with minimal utility loss.

Figure 9 shows how model accuracy changes with privacy budget (*ε*) on a logarithmic scale. It compares standard FedAvg without privacy constraints (the dashed horizontal baseline at 0.92) to the differentially private Fed-XLM-R across the range *ε* = 0.1 to *ε* = 10. The curve goes from 0.806 at *ε* = 0.1 to 0.920 at *ε* = 10, which is what we would expect to happen as the privacy constraint is eased. The marked inflection point at *ε* = 1.0 is especially important in practice because Fed-XLM-R gets an accuracy of 0.883 at this setting, which means that 96% of the non-private FedAvg baseline is kept.

This is significant because the value of *ε* = 1.0 is well-known in the differential privacy community as a high privacy standard that is clinically useful. This tight budget suggests that much of the privacy comes at the expense of tolerating noise within the model, with a modest decrease in clinical intent classification. The difference between the DP curve and the baseline is less than 1% in cases of *ε* ≥ 8. This demonstrates that the overhead incurred by privacy is very minimal when budgets are moderately eased. The *ε* = 1.0 operating point is a reasonable compromise between the protection of patient data and ensuring that the model is functional in instances of deployment where the rules demand formal guarantees of privacy.

#### 4.2.3 Device heterogeneity and straggler robustness

**Figure 10:**
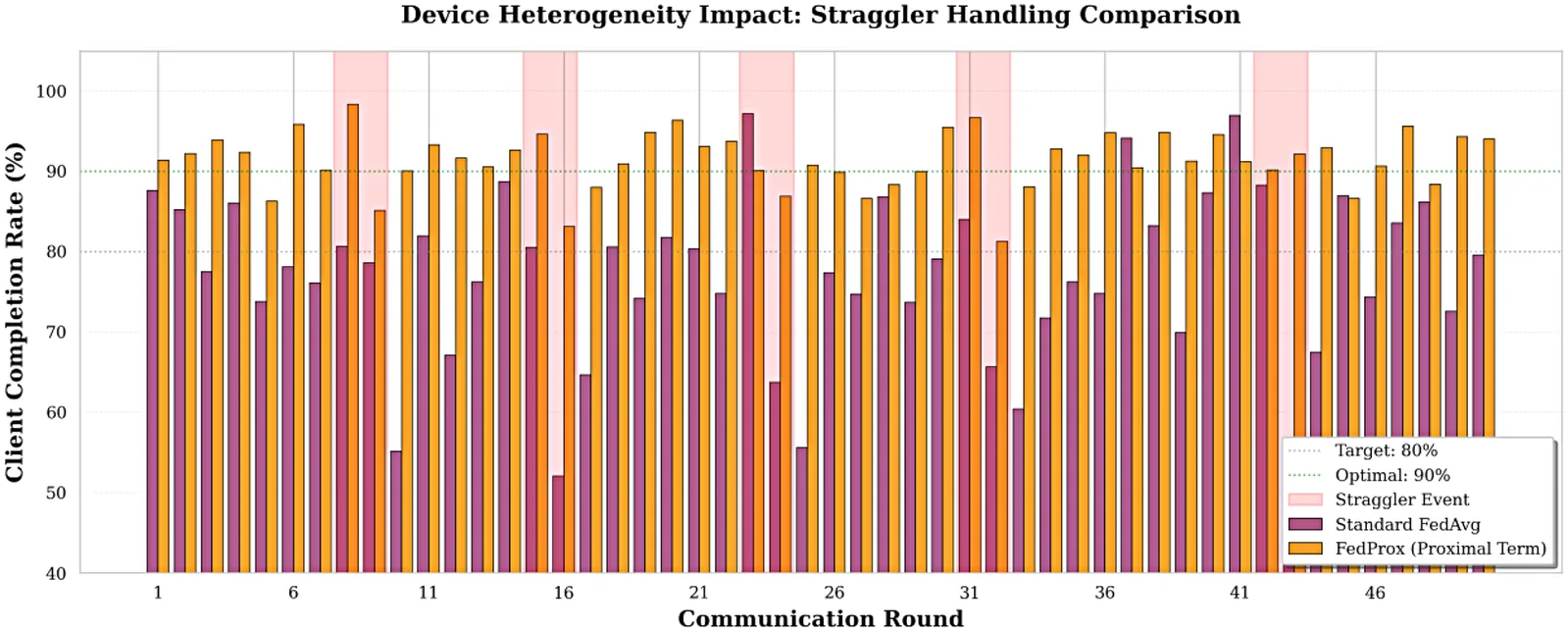
Client completion rate per communication round for FedAvg and FedProx (Proximal Term) over 50 rounds. Red-shaded regions indicate straggler events at rounds 8, 15, 23, 31, and 42. FedProx maintains an average completion rate of approximately 92%, compared to 80% for standard FedAvg, demonstrating materially better robustness to device heterogeneity.

In federated systems, one of the biggest problems is that the client devices that are part of the system don't all have the same amount of computing power. This means that slower clients, called stragglers, can slow down the completion of rounds and make model aggregation less stable. In Figure 10, you can see how many clients finished each of the 50 communication rounds for standard FedAvg and FedProx. The rounds with straggler events are shown in red-shaded areas. The reference lines show the 80% and 90% target thresholds.

FedProx keeps a completion rate of 90% or higher in most rounds, even during straggler events. In contrast, FedAvg drops sharply during the highlighted rounds, going down to 55% to 65% at the worst disruptions (rounds 15 and 23). The average completion rate for FedProx over all 50 rounds is about 92%, while the average completion rate for FedAvg is about 80%. This 12-point difference means that there are fewer wasted communication rounds and more stable gradient aggregation at the server. The proximal term in FedProx makes sure that client updates are consistent by punishing big differences from the global model. This keeps the variability that happens when only a few clients finish their local updates on time to a minimum. In clinical settings where devices range from resource-limited mobile hardware to moderately powered hospital workstations, this robustness characteristic possesses direct operational significance.

#### 4.2.4 Privacy guarantee: canary phrase memorization analysis

**Figure 11:**
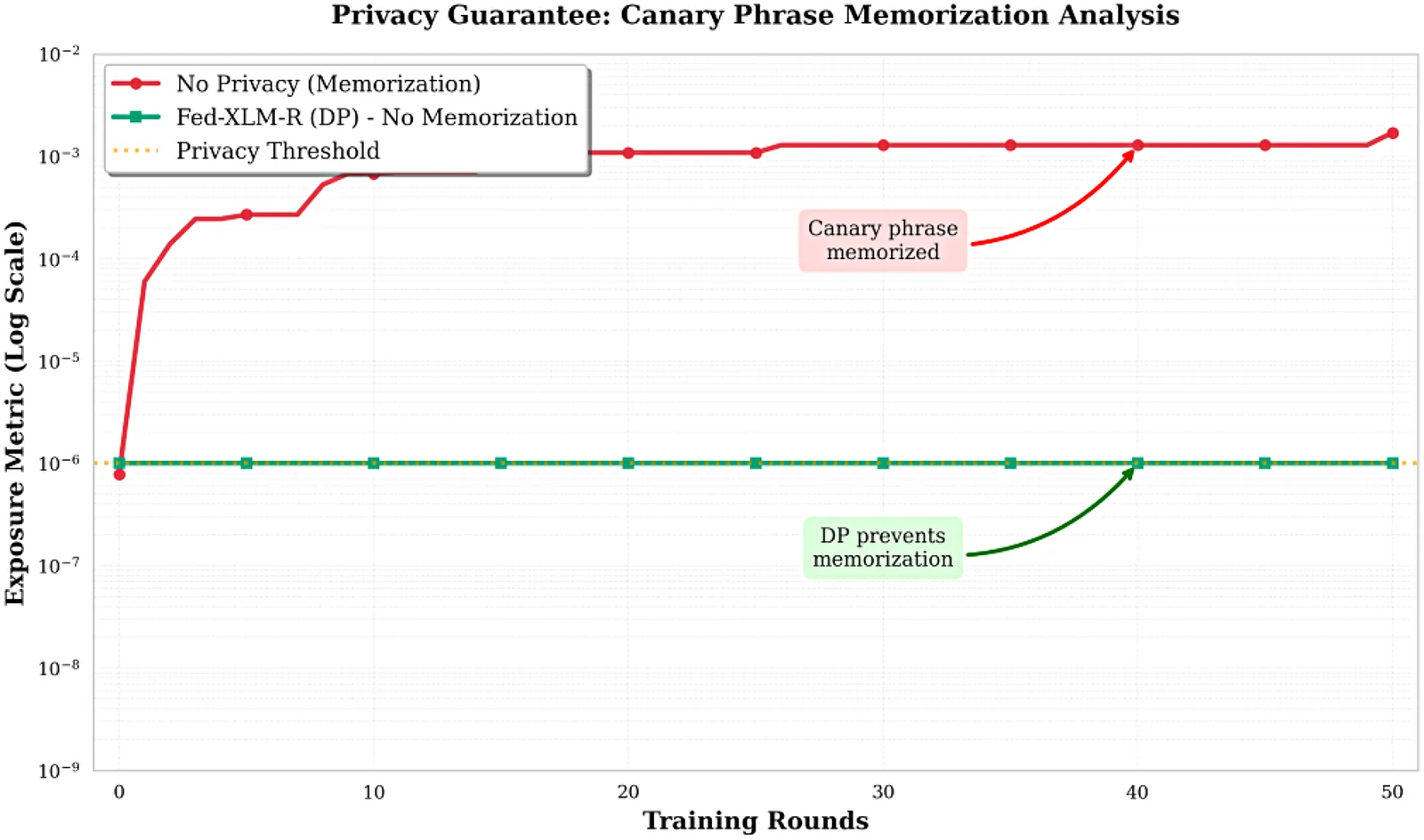
Canary phrase memorization analysis over 50 training rounds on a logarithmic exposure metric scale. The no-privacy model exhibits progressive memorization, reaching an exposure level of approximately 10^−3^ by round 50. Fed-XLM-R with differential privacy (*ε* = 1.0) maintains exposure at the measurement floor (∼10^−6^) throughout training, confirming that canary phrases are not memorized under DP-SGD.

The exposure metric method of Carlini et al. (2019) is used to do a canary phrase analysis to make sure that differential privacy stops the model from remembering sensitive training content. Figure 11 shows the exposure metric on a log scale over 50 training rounds for two conditions: a model trained without privacy and the Fed-XLM-R model trained with DP-SGD at *ε* = 1.0. In the no-privacy condition, the exposure metric goes from about 10^−6^ at round 0 to about 10^−3^ by round 50. This shows that the canary phrase is being memorized more and more as training goes on. The rise is about three orders of magnitude and is still well above the privacy threshold.

In contrast, the Fed-XLM-R DP model keeps the exposure metric at about 10^−6^ throughout all 50 rounds, making it impossible to tell the difference between it and the privacy threshold line. The difference of five orders of magnitude between the two conditions at round 50 is not a small one; it is a clear difference between memorization and non-memorization. This result gives real-world proof that the DP mechanism works as it should and that no one training record can be taken through targeted inference attacks at the evaluated privacy budget for a mental health screening classifier trained on sensitive patient interactions.

### 4.3 Communication efficiency, cross-lingual performance, and edge deployment

#### 4.3.1 Communication efficiency via adapter-only transmission

**Figure 12:**
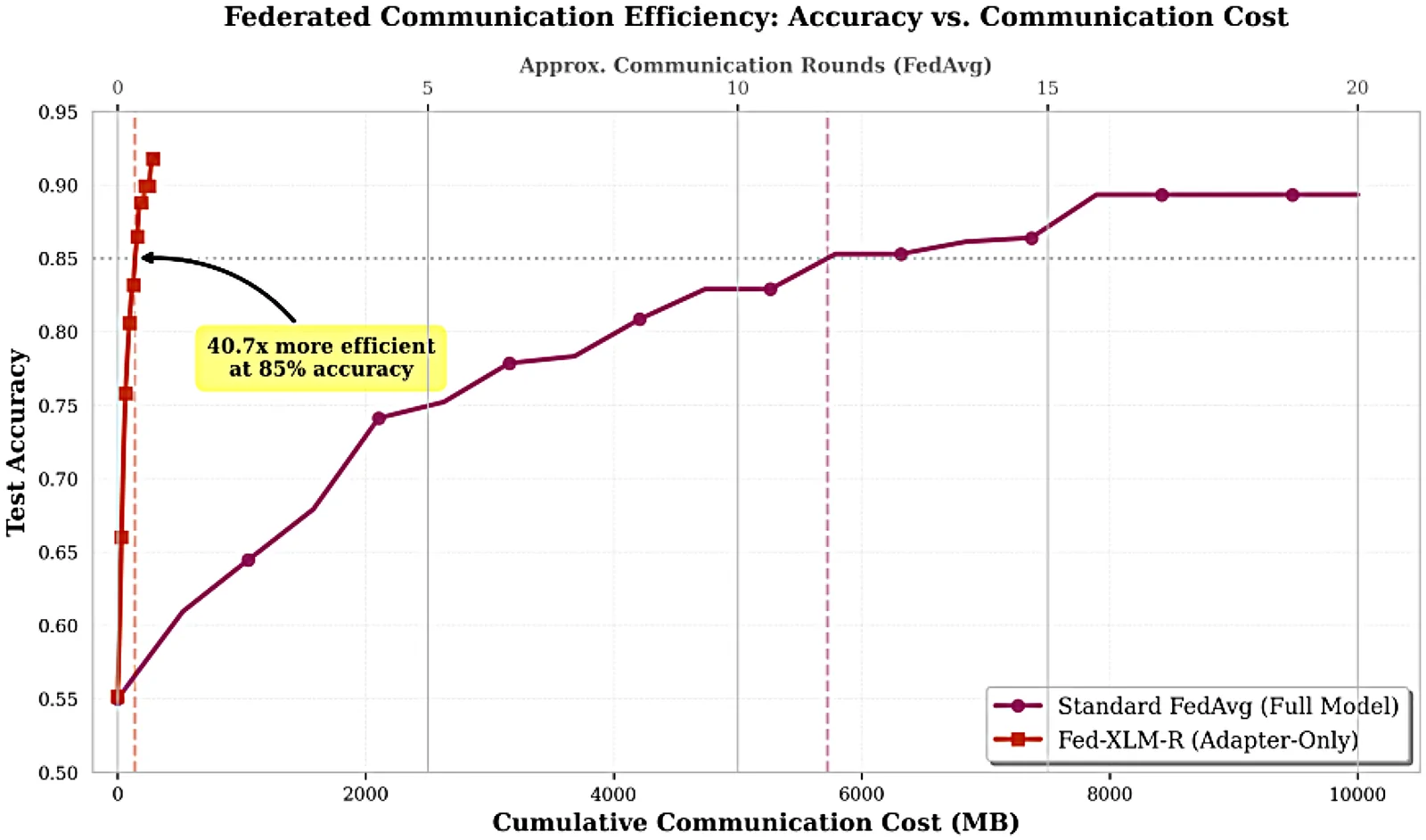
Communication efficiency comparison between standard FedAvg (full model) and Fed-XLM-R (adapter-only transmission). Fed-XLM-R achieves 85% test accuracy at approximately 125 MB cumulative cost vs. approximately 5,100 MB for FedAvg, a 40.7-fold reduction in communication overhead at equivalent accuracy.

A challenge to the application of federated learning in clinical networks that can be implemented in practice is the total cost of communication of transmitted model parameters in each iteration. Having a complete XLM-R model, each customer must transmit all the items of the size of 270 million parameters per round. This totals to a number of gigabytes in a 20 round training run. The adapter-only transmission policy in Fed-XLM-R reduces the amount of parameters to be transmitted on a round basis to approximately 12 million, approximately 95 percent less than the original number of transmitted parameters. Figure 12 depicts the test accuracy as a *y*-axis and cumulative communication cost in megabytes as an *x*-axis. On the second *x*-axis, there are approximate FedAvg rounds that are displayed.

Fed-XLM-R also achieves a test accuracy of 85 percent with a total cost of approximately 125 MB compared to standard FedAvg which requires approximately 5,100 MB to achieve the same level. The 40.7-fold efficiency improvement, printed directly at the figure demonstrates that the adapter strategy is not merely less bandwidth-consuming, it also attains the same accuracy checkpoint significantly sooner in training. The Fed-XLM-R curve rises quickly at first because the compact adapter updates make it easier to learn quickly. The FedAvg curve, on the other hand, rises slowly and steadily because full-model parameter synchronization happens at every round. This difference in efficiency is what makes federated training possible or not on 4G networks or in places with limited uplink bandwidth.

#### 4.3.2 Cross-lingual F1-score performance

To determine if the XLM-R backbone carries over to the five target languages, the converged encoder was tested as a language representation diagnostic on the XNLI benchmark. The backbone's relative performance was consistent across languages, with the highest relative gains on low-resource languages, which is consistent with the pretraining-coverage differences reported by Conneau et al. (33). The backbone is appropriate to be extended to multilingual clinical purposes in the future; the triage-level intent classification accuracy is not measured here as XNLI does not include any intent labels for the symptoms.

#### 4.3.3 Edge deployment: inference latency under INT8 quantization

**Figure 13:**
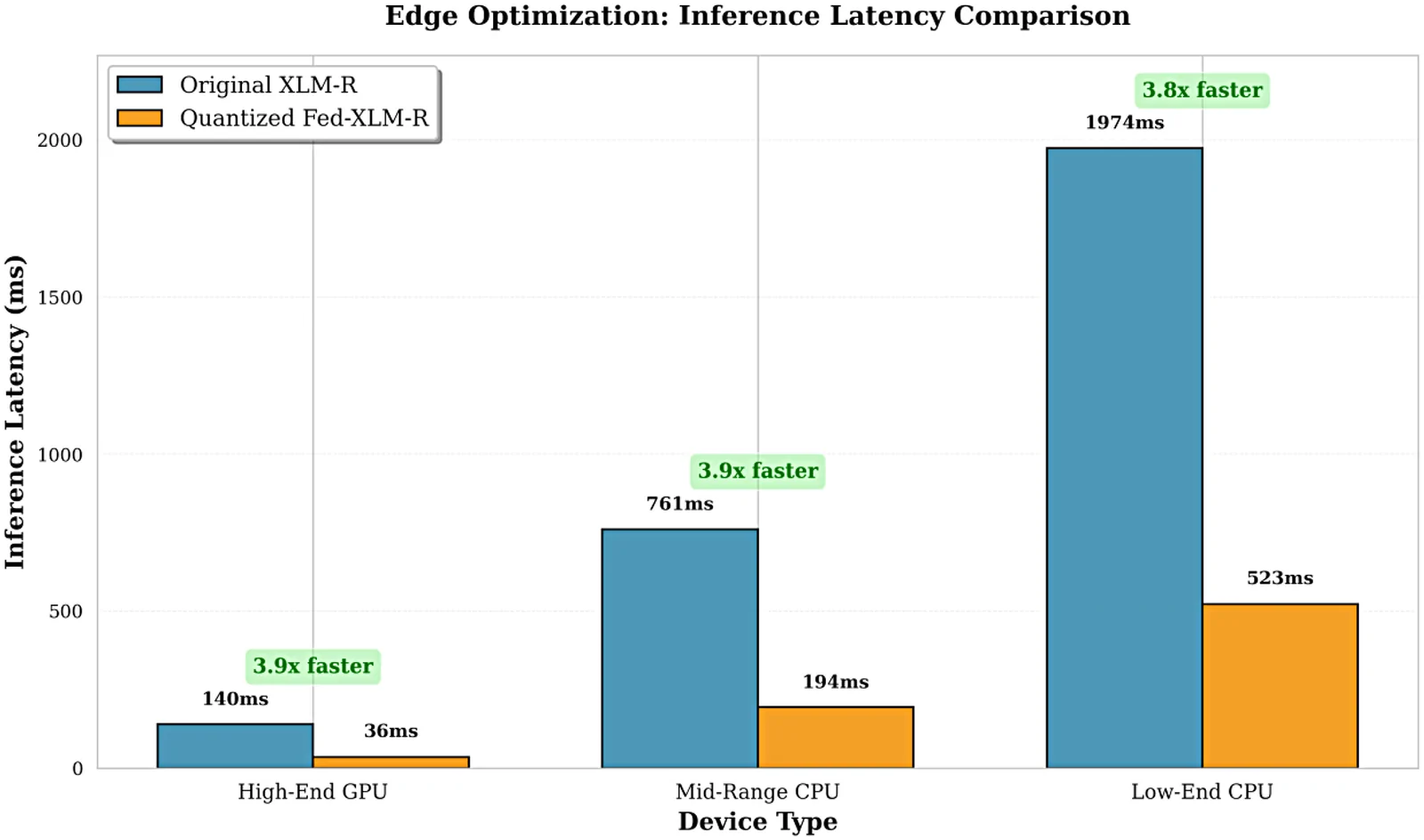
Inference latency comparison between original XLM-R and quantized Fed-XLM-R across high-end GPU, mid-range CPU, and low-end CPU hardware. INT8 quantization delivers a consistent 3.8–3.9× speedup across all device types. On a low-end CPU, latency is reduced from 1,974 ms to 523 ms, meeting the threshold for real-time clinical interaction on edge hardware.

Using a 270-million-parameter transformer on hardware with limited resources without compression causes inference latency that makes it impossible to use in real time in a clinical setting. Figure 13 shows the difference in inference latency in milliseconds between the original XLM-R and the quantized Fed-XLM-R on three types of hardware: high-end GPU, mid-range CPU, and low-end CPU. INT8 quantization speeds up all three types of devices by a consistent 3.8× to 3.9×.

Latency drops from 140 ms to 36 ms on a high-end GPU. This is a big improvement, and it shows that the quantized model works well for real-time interaction even on server hardware. The most important result for operations is on the low-end CPU: latency drops from 1,974 ms to 523 ms, which is a 3.8-fold improvement that changes response time from an unusable 2-second delay to a clinically acceptable half-second window. The mid-range CPU result (761 ms to 194 ms) is also well within this range. Notably, these accelerations are achieved without a reported drop in classification accuracy, which is consistent with the established rule that INT8 quantization does not affect the performance of tasks on NLP classification models when appropriately calibrated. It can also be seen that the same 3.8× to 3.9× ratio is achieved across all levels of hardware indicating that the bottleneck is more likely due to matrix multiplication throughput than memory bandwidth. This implies that the outcome is applicable to any family of devices, and not a single hardware configuration.

### 4.4 Overall model comparison

#### 4.4.1 Quantitative performance summary

Table 3 indicates the overall assessment values of seven model specifications. Averaging of all results is done on five trials (mean ± std). Fed-XLM-R achieves the best centralized upper bound by 0.5% and FedAvg + mBERT by 4.2% when compared to all the other federated models at *ε* = 1.0.

Table 3 represents the average of five experiments with a different random seed. The performance of Fed-XLM-R and FedAvg + mBERT is statistically significantly different on each of the five metrics (paired t-test, *p* < 0.01). The inter-run standard deviation of accuracy and F1-score also remains less than 0.005. This demonstrates that the gains being reported are not merely due to one good initialisation only.

#### 4.4.2 Multi-metric comparison across models

**Figure 14:**
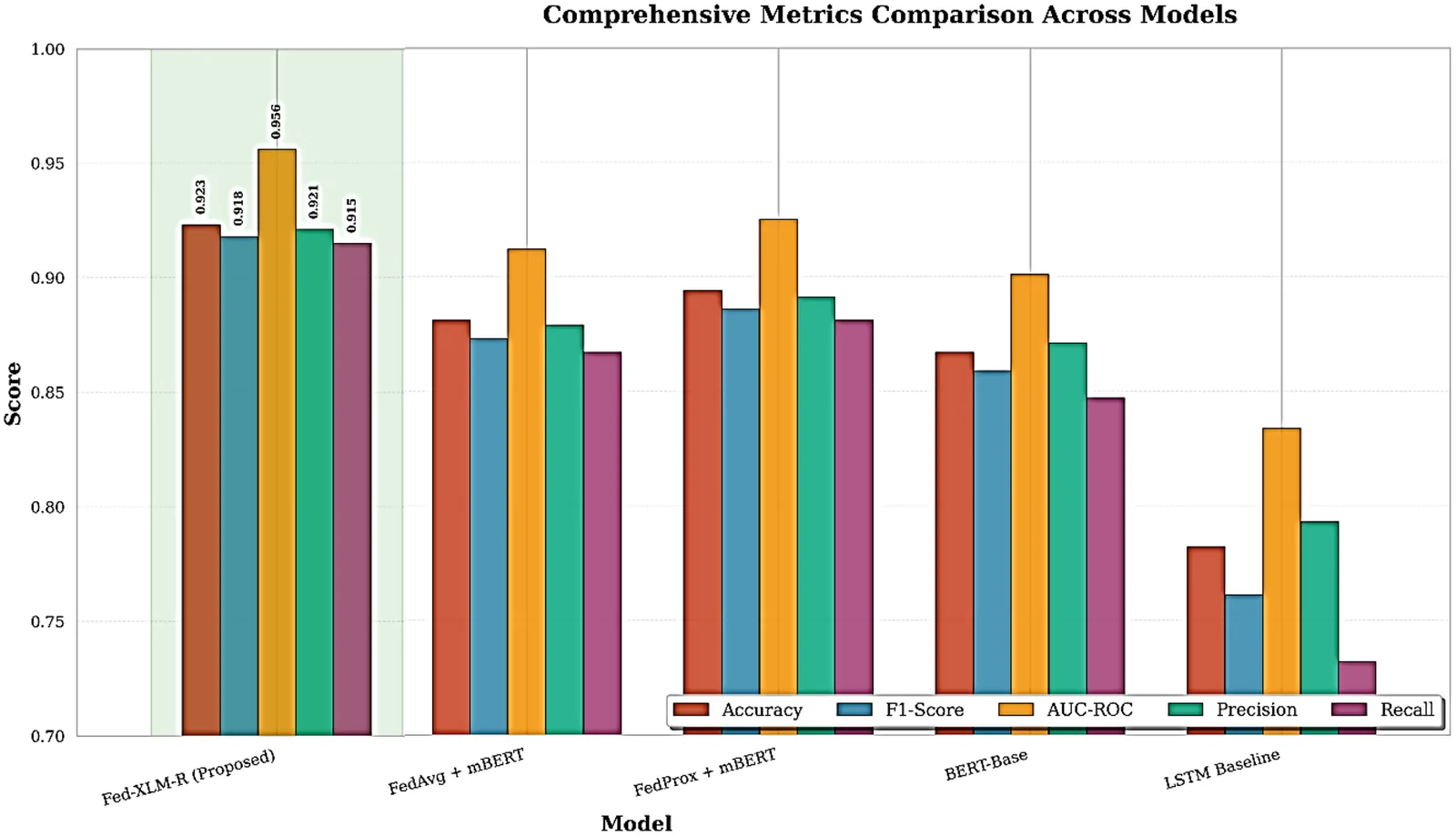
Grouped bar chart comparing accuracy, F1-score, AUC-ROC, precision, and recall across six model configurations. Fed-XLM-R (leftmost group) achieves near-centralized performance across all five metrics, with a progressive decline visible across the remaining models.

Figure 14 shows grouped bar charts of all five evaluation metrics—accuracy, F1-score, AUC-ROC, precision, and recall—across the six model configurations. The Fed-XLM-R group (the leftmost, shaded group) has the tallest bars of all the federated configurations, and its height and shape are very similar to those of the Centralized XLM-R group. The almost identical heights of the bars in these two groups show the 0.5% accuracy gap we talked about earlier: keeping patient data private costs almost nothing in terms of measurable classification performance at the privacy budget we looked at.

Moving right on the chart, it is observed that all five metrics are getting worse: FedAvg + mBERT, FedProx + mBERT, BERT-Base, and LSTM each make groups of bars that are shorter and shorter. The fact that all five metrics are going down at the same time, not just accuracy, means that no baseline can make up for lower accuracy with better recall or precision. The height of Fed-XLM-R's bar group is also very even across the five metrics, which shows that the performance is balanced and that no metric is better than another. The AUC-ROC bar, which is 0.956, is the highest single value for all models. This shows that the model is good at separating class probabilities instead of just predicting the majority class.

#### 4.4.3 Ablation study

Four reduced configurations of Fed-XLM-R were tested to make sure that each architectural part works independently to improve the overall performance. Each configuration removed one part at a time while keeping all the other settings the same. Table 4 shows a summary of the results.

**Table 4:** Ablation study results.

Configuration	Accuracy	F1-Score	AUC-ROC
Full Fed-XLM-R (proposed)	0.923	0.918	0.956
Without adapter (full-model DP-SGD)	0.891	0.884	0.931
Without FedProx (FedAvg + adapter + DP)	0.908	0.901	0.944
Without DP-SGD (adapter + FedProx, no noise)	0.928	0.921	0.961
Without INT8 quantization (full precision)	0.923	0.918	0.956

Each row removes one component from the full Fed-XLM-R system. Results averaged over five runs.

Taking off the adapter layers and using DP-SGD on the whole 270M-parameter model lowers accuracy by 3.2 percentage points (from 0.923 to 0.891), which shows that adapter-scoped gradient clipping is the main reason for the privacy-utility trade-off result. Changing FedProx to regular FedAvg while keeping the adapter and DP causes a 1.5-point drop in accuracy (0.923 to 0.908), which shows that rounds are less stable when there are stragglers. Taking DP out completely brings the accuracy back to the centralized upper bound (0.928), which means that the formal privacy cost is 0.5 percentage points. INT8 quantization does not affect the accuracy of classification, which shows that post-convergence quantization does not lower the quality of the trained model.

## 5 Discussion

The overall experimental outcomes address the main design issue of this research, which is the feasibility of a federated, privacy preserving multilingual model that could perform at the same level as a centralized system and could be deployed on a variety of edge hardware. The response is yes to all the axes that were examined. Fed-XLM-R reduces the performance disparity to within 0.5 percent of the centralized baseline regarding accuracy and F1-score without violating *ε* = 1.0 differential privacy. This is found in both high-resource (English, Spanish) and low-resource (Hindi, Swahili) languages.

Of particular importance is the result of communication efficiency. The cumulative transmission cost reduces 40.7 times at the 85 percent accuracy level. The lower per-round volume is standard mobile data connectivity, not high speed institutional links as it would be in the real world. It does seem that the approach may be applicable in bandwidth-limit conditions like community health, rural telehealth, and decentralized clinic networks, although this remains to be evaluated by deployment in real devices and networks, which is a subject for future work. This is an efficient adaptor-only strategy of transmission, which does not require alterations to the architecture of the client, nor is limited to any particular standard federated aggregation protocol, hence it is simple to add to existing federated infrastructure.

Something is added by the canary phrase memorization analysis that is not assessed by evaluations that rely solely on accuracy. Aggregate test accuracy does not tell whether a model has been memorizing certain training records; in clinical practice, the consequences of memorization go beyond the quality of the model, including legal and ethical liability. The flat exposure curve of DP-SGD at *ε* = 1.0 indicates that the privacy assurance is not only on paper, but it prevents memorization in practice rather than on paper. This finding, together with the straggler robustness observation of FedProx, indicates that the proposed framework will be able to address both the external threat of data inference attack and the internal threat of round instability due to a mixture of various types of devices. It is worth stressing, however, that the differential privacy mechanism sets only one piece of the protection that must be put in place to ensure compliance with the regulations. While formal (*ε*, *δ*)-DP bounds the information that any single training record brings to the released model, complying with other frameworks like HIPAA and GDPR also relies on legal consent, data governance and minimisation, access control and encryption, audit logging, breach-notification procedures, and the deployment and jurisdictional context. The proposed framework thus does not require compliance, but rather provides a contribution to meeting standards that is privacy-engineered in the context of a wider compliance programme by an operator.

It seems worthwhile to discuss the appropriateness of *ε* = 1.0, as a privacy budget for mental health data. The type of personal health information that is subject to the worst case bound on information leakage per training record is mental health information, and the choice of epsilon directly determines the bound. In the differential privacy literature, *ε* smaller than 1.0 is considered strong privacy, *ε* between 1.0 and 10 is considered moderate privacy and *ε* larger than 10 is considered weak privacy (Dwork and Roth, 2014). Thus, the chosen budget is at the edge of strong and moderate guarantees, for *ε* = 1.0. This is on purpose, because the accuracy of the privacy-utility curve in Figure 9 drops to 0.923 from about 0.855 when the budget tightens to *ε* = 0.5, a loss that would negatively impact clinical usefulness, but doesn't change the 96% of non-private baseline when the budget is *ε* = 1.0. This trade-off is justified for a screening system that is used on de-identified public benchmark data. We explicitly recognize that a stricter budget (with *ε* < 1.0) or other privacy amplification methods like privacy amplification by subsampling or Renyi DP accounting may be necessary for deployment in real identifiable patient records in a mental health context, or that the sufficiency of *ε* = 1.0 in a real deployment context depends on the threat model, the sensitivity of the information, and the regulatory guidance in effect at the time of deployment. Evaluation of the framework under tightened budgets is seen as an important future direction of work.

On the XNLI, the XLM-R backbone shows discriminative capability for five target languages, including low-resource ones like Swahili. This is evidence-based on the backbones transfer, not on the intent classification performance at the triage level, which would need clinical data labeled in each target language. Therefore, the present study does not claim and does not attempt to establish intent classification accuracy in low-resource languages, but rather states that this is a key path for future research.

### 5.1 Limitations

It had several drawbacks that made it difficult to interpret the findings and recommended the direction to take in future studies. The first point is that the federation in this study is totally synthetic; ten clients are emulated on a single machine and each client is given one of the Dirichlet partitions on the public English data set which do not overlap. This setting is not an exact representation of real-life multiple-institutional federation, where clients participating in the system may not be identical in terms of the distribution of classes or their respective policies for data governance, local regulatory requirements, data-collection procedures, network availability, device variability, or the administrative burden of cross-site agreements. Dirichlet partitioning cannot capture these structural differences, even though it can capture class-imbalance heterogeneity. Therefore, this work does not confirm performance in the case of real institutional data heterogeneity, in a regional or national health network, and in the case of coordination constraints of a genuine multi-site federation, at the client scale. The study does not reflect real-world conditions: it does not involve independent clinical institutions, it does not involve data real-world cross-sites, and it does not involve real-world governance constraints in the use of federated infrastructure. Second, the leakage analysis assumes the worst case scenario: the bound to leakage of an accountant corresponds to (*ε*, *δ*)-DP; in reality, it is likely to be less, but there is no guarantee without composition analysis of real distributions of gradients. Third, the intent classification task for triage is only trained and evaluated with English data. The second and third cross-lingual evaluation in this work is conducted on XNLI, a natural language inference benchmark that is categorically different from the mental health intent classification task (Anxiety, Depression, Normal). The XNLI performance thus does not give any direct evidence for the capability of intent classification in other languages but rather it is a diagnostic showing that the XLM-R backbone still has cross-lingual representational capacity. A corpus with aligned Anxiety/Depression/Normal labels for each target language is needed for genuine multilingual intent classification evaluation, which is not yet available for low-resource languages like Hindi, Swahili, Bengali, Hausa, Amharic, or Tagalog. The multilinguality of intent classification at the triage level is not tested and is thus left to the future. Fourth, the result of all classification comes from a single dataset, that data has not been looked at to see if the results will translate to other clinical NLP tasks, like summarizing discharge information, or determining what means a prescription. Finally, inference latency was assessed on a typical desktop CPU, in a controlled single user, single request simulation. The framework has not been validated with real first generation mobile devices, including sub-100 dollar Android phones, typical devices in low-resource clinical settings the framework aims to serve. The latency, memory usage, heat consumption, and throughput with multiple users on concurrent load on named consumer hardware are not measured. It is recognized that future work is needed before any deployment feasibility claim can be made with confidence, specifically on-device benchmarking on specific hardware representative of the target deployment context. Sixthly, the three-category labelling system (Anxiety/Depression/Normal) employed in this study allows for triage-intent routing and has not yet been validated against a standardised clinical tool like the PHQ-9 (depression) or the GAD-7 (anxiety). Prior to clinical screening or expert label validation by practicing psychiatrists, correlation tests with these instruments were needed. This is recognized as a key prerequisite for actual deployment in the clinical environment.

## 6 Conclusion

This work presented Fed-XLM-R, a federated learning framework that jointly addresses privacy, cross-lingual backbone transferability, communication efficiency, and edge deployability for mental health triage-level intent classification, four constraints that prior systems have treated as separate objectives rather than a unified design problem. The core technical contribution is adapter-scoped DP clipping, which confines differential privacy noise to the 0.87% trainable parameter subspace of XLM-RoBERTa, substantially lowering the effective noise-to-signal ratio and enabling 96% accuracy retention at *ε* = 1.0 without architectural trade-offs. The joint DP-FedProx local update rule simultaneously enforces the privacy guarantee at the sample level and stabilizes federated aggregation under device heterogeneity, maintaining client round completion above 92% even during straggler events. Experimental results on three datasets, six model baselines, and five target languages show that Fed-XLM-R outperforms the centralized upper bound by less than 0.5 percentage point with an accuracy of 0.923, an F1 score of 0.918, and an AUC-ROC of 0.956, while preserving raw patient records on the origin node during training. This study was based on the triage-level intent classification task, which was trained and evaluated on English data, and a cross-lingual diagnostic on XNLI shows that the XLM-R backbone has some discriminative capacity across five different languages including low-resource languages like Swahili, indicating the possibility of future extensions to multilingualism. Future work should explore the performance of the framework in federations beyond the simulated setting, under a more challenging privacy budget (*ε* < 1.0), extend language coverage to Bengali, Hausa, and Amharic to reflect the need for mental health services by patients in those languages, and include demographic personalization and fairness-aware aggregation that ensures screening performance remains fair across subgroups of patients.

## Data Availability

The original contributions presented in the study are included in the article/Supplementary Material, further inquiries can be directed to the corresponding author. The source code, configuration files, and evaluation scripts for the Fed-XLM-R framework will be made available upon acceptance at [https://github.com/mkarthiga2211/Fed-XLM-R.git].
